# An integrated nano-delivery platform via baicalein-loaded polydopamine-MOF for enhanced neuroprotection against retinal ischemia reperfusion injury

**DOI:** 10.1016/j.mtbio.2026.103239

**Published:** 2026-05-21

**Authors:** Xin Liu, Keke Huang, Zhiqing Lin, Min Tang, Qianyi Lin, Wangdu Luo, Jiaguo Yuan, Junlong Yu, Yujie Rao, Peizeng Yang, Lin Xie

**Affiliations:** a Department of Ophthalmology, The Third Affiliated Hospital of Chongqing Medical University, Chongqing, 401120, China; b Chongqing Key Laboratory for the Prevention and Treatment of Major Blinding Eye Diseases, Chongqing, 400042, China; c Department of Ophthalmology, Affiliated Hospital of Southwest Jiaotong University, The Third People's Hospital of Chengdu, Chengdu, 610031, China; d Department of Ophthalmology, West China School of Medicine, Sichuan University, Sichuan University Affiliated Chengdu Second People's Hospital, Chengdu Second People's Hospital, Chengdu, 610031, China

**Keywords:** Retinal ischemia reperfusion injury, Polydopamine, MOF, Retinal ganglion cells, Oxidative stress

## Abstract

Retinal ischemia reperfusion injury (RIRI) presents a complex pathological mechanism involving oxidative stress, neuroinflammation, and retinal ganglion cell (RGC) apoptosis, with current therapies limited by their inability to address these multifaceted cascades. To address this challenge, we engineered microenvironment-responsive nanoparticles, pZIF-8@Bai, by encapsulating baicalein (Bai) within a polydopamine-coated zeolitic imidazolate framework (ZIF-8), The pZIF-8@Bai nanoparticles achieved targeted combination therapy through a sustained-release mechanism, resulting in comprehensive retinal protection: attenuating oxidative damage, promoting microglial M2 polarization, and significantly inhibiting RGC apoptosis. Importantly, functional assessments demonstrated substantial recovery of a-wave and b-wave amplitudes in electroretinography (ERG) and enhanced P1-wave amplitudes in flash visual evoked potential (FVEP) recordings. Mechanistically, proteomic analysis revealed suppression of Nogo-A expression, which modulated NF-κB signaling and the intrinsic apoptotic pathway, involving caspase-8 inhibition and BCL-2/BAX balance restoration. These actions preserved retinal structural integrity and restored visual function, as confirmed by electroretinography, with excellent biosafety profiles, offering a promising multi-target therapeutic strategy for neurodegenerative retinal diseases.

## Graphical abstract

**Figure:**
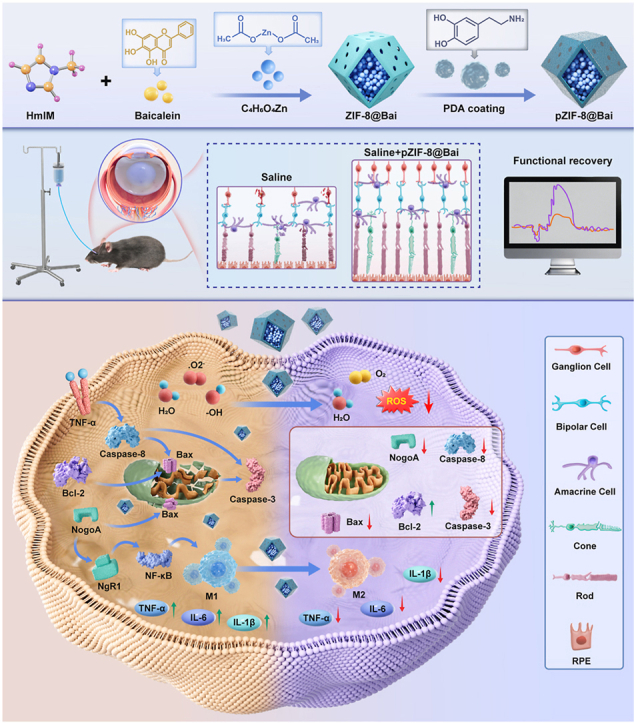

## 1 Introduction

Retinal ischemia reperfusion injury (RIRI) represents a critical pathophysiological process underlying a spectrum of vision-impairing ophthalmic conditions, including glaucoma, diabetic retinopathy, and retinal vascular occlusions [1]. The injury cascade is characterized by an initial ischemic insult resulting from circulatory interruption, followed by a pronounced secondary damage during reperfusion—a phenomenon often described as a “second hit” that exacerbates cellular dysfunction and tissue loss [[2], [3], [4]]. Epidemiological evidence indicates that ocular diseases associated with RIRI impose a substantial global disease burden. According to 2020 statistical data, three major blinding eye diseases—glaucoma, diabetic retinopathy, and age-related macular degeneration—collectively caused over 19 million cases of moderate or severe visual impairment worldwide in people aged 50 years and above [5]. With the accelerating trend of global population aging, this number is expected to continue rising, presenting significant challenges for preventing and treating RIRI-related eye diseases.

RIRI involves a multifaceted and tightly regulated pathological cascade. The ischemic phase is primarily characterized by cellular energy metabolism dysfunction [6], ion homeostasis imbalance [7], and cell membrane depolarization [8]. The reperfusion phase, through sudden reoxygenation, generates excessive reactive oxygen species (ROS), which subsequently activate crucial inflammatory signaling pathways such as Toll-like receptor 4/nuclear factor kappa-B (TLR4/NF-κB) [9]. This promotes microglia/macrophage polarization toward the pro-inflammatory M1 phenotype, initiating intense inflammatory responses [10]. These pathological alterations collectively disturb neuroimmune homeostasis, compromise blood-retinal barrier integrity, and drive progressive retinal ganglion cell (RGC) apoptosis. Notably, a positive feedback loop forms between oxidative stress and neuroinflammatory responses, creating a vicious cycle that continuously amplifies the damage [11]. Faced with a growing clinical burden, current therapeutic strategies for RIRI-associated ocular diseases remain substantially limited. Mainstay interventions—such as intraocular pressure management in glaucoma and *anti*-VEGF therapies in diabetic retinopathy—predominantly target singular pathological pathways, failing to address the interconnected mechanisms underlying RIRI. Furthermore, ocular drug delivery is significantly hindered by the blood-retinal barrier and other anatomical constraints, resulting in poor bioavailability, short half-lives, and the need for repeated invasive administrations [[12], [13], [14]]. Consequently, developing innovative treatment strategies that can effectively traverse ocular biological barriers and achieve multi-target synergistic intervention has become an urgent necessity and research priority for addressing the clinical challenges of RIRI-related eye diseases.

*Scutellaria baicalensis*, a plant used in traditional Chinese medicine, contains flavonoids as the primary active compounds extracted from its dried roots, including baicalein (Bai) and baicalin [15,16]. Studies have demonstrated that Bai exhibits notable antioxidant and anti-inflammatory properties, effectively suppressing the generation of ROS such as superoxide (O_2_^−^) and hydrogen peroxide (H_2_O_2_), while also strongly inhibiting iron-dependent lipid peroxidation [17,18].In experimental models of ischemia-reperfusion (I/R) injury, Bai attenuates oxidative stress and exerts neuroprotective effects, partly through upregulation of heme oxygenase-1 (HO-1) and downregulation of pathways related to hypoxia-inducible factor-1α (HIF-1α), vascular endothelial growth factor (VEGF), and matrix metalloproteinase-9 (MMP-9) [19]. Additionally, Bai has been found to inhibit aldose reductase activity and exert a dose-dependent protective effect on oxidative stress-induced damage in RGC, indicating its potential value in retinal diseases such as glaucoma [20]. Despite these promising pharmacological properties, the clinical translation of Bai is hampered by considerable delivery challenges. Although intraocular injection can achieve localized high drug concentrations, the short half-life and low bioavailability of Bai limit the sustained presence of therapeutic concentrations at the target site. Moreover, conventional formulations are unable to achieve controlled drug release due to the eye's unique anatomical and physiological barriers, such as the blood-retinal barrier, which considerably limits treatment efficacy. Therefore, developing novel delivery systems—such as nanocarriers or sustained-release formulations—capable of enhancing retinal penetration and enabling prolonged pharmacological activity represents a critical direction for improving the clinical utility of Bai in retinal diseases.

To address the aforementioned limitations, we designed and synthesized a novel nano-synergistic therapy platform: polydopamine-coated metal-organic framework material Zeolitic Imidazolate Framework-8 (ZIF-8) loaded with Bai (pZIF-8@Bai). The design rationale is based on several synergistic considerations: ZIF-8, a subclass of metal–organic frameworks, offers a high surface area and tunable porosity, facilitating high-capacity encapsulation of the hydrophobic drug Bai and enhancing its aqueous stability and dissolution behavior [21,22]. The polydopamine (PDA) shell providing multiple synergistic functions that confers several therapeutic advantages. Firstly, PDA itself is an efficient ROS scavenger, capable of directly neutralizing excessive reactive oxygen species such as superoxide anions and hydroxyl radicals produced during the early stages of reperfusion, thereby curbing the initiation of oxidative stress at the source and forming a first layer of synergy with the antioxidant effects of Bai [23,24]. Secondly, by mitigating ROS, PDA indirectly suppresses downstream pro-inflammatory signaling pathways such as TLR-4 [25]， Thereby alleviating the subsequent inflammatory storm. Furthermore, the PDA coating exhibits good bio adhesion [26], which can prolong the retention time of the nanoparticles (NPs) within the eye. Simultaneously, it acts as a physical barrier, modulating the release rate of Bai to avoid burst release and achieve sustained therapy.

**Scheme 1:**
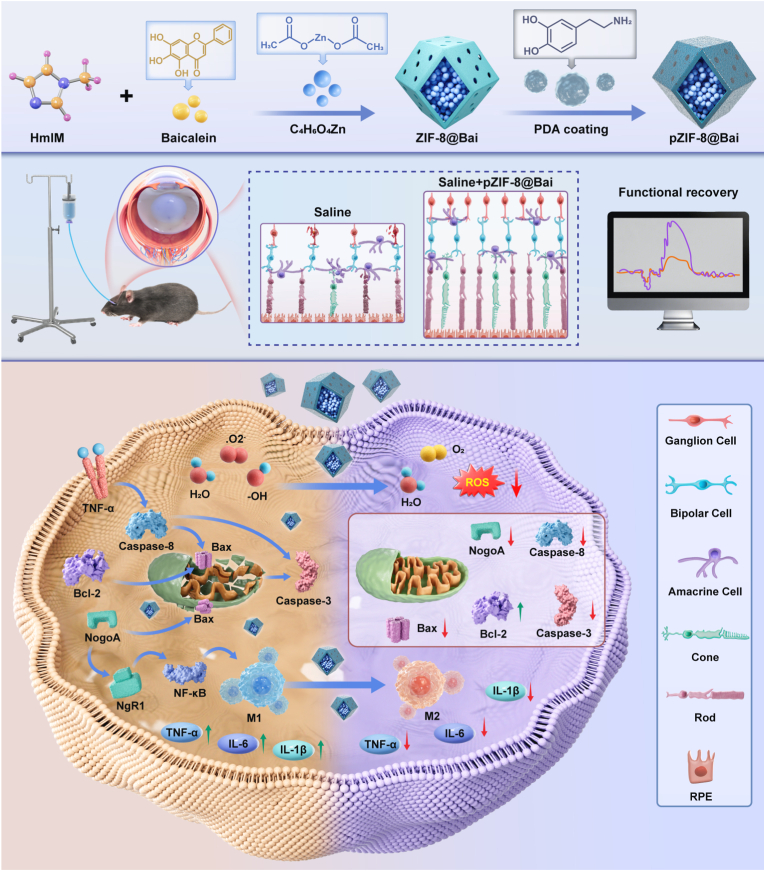
Schematic illustration of the synthesis of pZIF-8@Bai NPs as a novel drug delivery system, demonstrating its mechanism in providing visual impairment protection and therapeutic effects for retinal ischemia reperfusion injury by attenuating oxidative damage, promoting microglial M2 polarization, and significantly inhibiting retinal ganglion cell apoptosis.

In the murine model of RIRI, intravitreal injection of the pZIF-8@Bai NPs demonstrated remarkable therapeutic efficacy through its sophisticated dual-targeting mechanism. The NPs leverage their nanoscale dimensions and the inherent bio adhesive properties of the polydopamine (PDA) coating to establish a physical barrier. Complete release of the drug molecules necessitates diffusion or progressive degradation/swelling of the PDA layer. Consequently, this mechanism converts the rapid initial “burst release” into a more gradual and prolonged release profile, thereby facilitating the maintenance of effective therapeutic concentrations within retinal tissues. Upon encountering the weakly acidic microenvironment characteristic of ischemic-reperfusion injury sites, the ZIF-8 core underwent rapid degradation, facilitating burst release of high-concentration Bai. This process initiated a coordinated therapeutic cascade: the PDA shell functioned as a primary defense by directly neutralizing ROS, while the liberated Bai provided a secondary protective effect through further attenuation of oxidative stress, downregulating key inflammatory factors (IL-6, TNF-α, IL-1β), and directly inhibiting RGC apoptotic pathways. *In vivo* animal study results revealed that pZIF-8@Bai administration significantly preserved retinal histological architecture, reduced TUNEL-positive apoptotic cells, and markedly increased Brn3a-positive RGC survival compared to controls. Immunofluorescence staining revealed suppressed microglial activation, as indicated by decreased IBA1 expression, alongside diminished IL-1β immunoreactivity. Functional assessments demonstrated substantial recovery of a-wave and b-wave amplitudes in electroretinography (ERG) and enhanced P1-wave amplitudes in flash visual evoked potential (FVEP) recordings. Proteomic analysis further identified downregulation of Nogo-A and related apoptotic signaling pathways (Scheme 1). The synergistic anti-oxidant and anti-inflammatory effects, combined with this microenvironment-responsive release mechanism, collectively validated our platform's efficacy in achieving multi-stage, multi-target therapy for RIRI.

## 2 Experimental section

### 2.1 Synthesis and characterization

The synthesis of pZIF-8 NPs was adapted from an established protocol with modifications [27]. Specifically, 0.1 M zinc acetate dihydrate (Zn(CH_3_COO)_2_·H_2_O) and 1.6 M 2-methylimidazole (HmIM) were co-dissolved in 20 mL of Tris-HCl buffer (pH 8.5). After brief agitation for 1 min, dopamine (DA) was introduced at a concentration of 0.5 mg/mL, and the reaction was maintained under continuous agitation at 150 rpm and 37 °C for 2 h. The resulting product was isolated by centrifugation at 10,000 rpm for 5 min, followed by three washing cycles with deionized water. The collected precipitate was subsequently lyophilized to yield the final pZIF-8 nanoscale metal-organic framework.

For drug loading, Bai was incorporated in situ during the crystallization process. Specifically, a Bai solution (10 mg/mL) was introduced into the precursor mixture containing Zn(CH_3_COO)_2_·H_2_O and HmIM prior to the addition of DA. This sequential addition strategy enabled the progressive encapsulation of Bai molecules within the growing ZIF-8 framework, ultimately yielding pZIF-8@Bai nanocomposites. The *in vitro* release of Bai from pZIF-8@Bai was evaluated by dispersing 1 mL of the sample into 5 mL of PBS buffer (pH 7.4 and pH 6.5), followed by incubation at 37 °C and 100 r/min in a shaking incubator. At designated time points, the released baicalein was quantified via UV-Vis spectrophotometry.

The morphological characteristics of the NPs were examined using TEM and SEM. Particle size distribution and surface charge were determined through DLS measurements performed on a Malvern Zetasizer Nano ZS system.

Elemental composition analysis was conducted via EDX. Additionally, the drug encapsulation efficiency was quantitatively assessed for both ZIF-8 and pZIF-8 nanoparticle formulations. Crystal phase identification of ZIF-8 and pZIF-8 nanoparticle was performed by X-ray diffraction (XRD, X'pert PRO, Philips, The Netherlands). Meanwhile, Fourier-transform infrared (FT-IR) spectra of specific amounts of Bai, PDA, ZIF-8, pZIF-8, and pZIF-8@Bai were collected on a Nicolet spectrometer (Thermo Fisher, USA) using potassium bromide (KBr) pellets.

### 2.2 Cell culture and treatment with pZIF-8@Bai

R28 cells (RRID: CVCL 5H51) were a gift from the Department of Anatomy and Neurobiology, Central South University. The BV2 microglial cell line (RRID: CVCL0182) was purchased from Shang En Biotechnology Company (Wuhan, China). R28 cells and BV2 microglial cells were cultured in DMEM (Gibco), supplemented with 10% fetal bovine serum (Lonzera, China) and 1% penicillin/streptomycin (Procell, China) at 37 °C with 5% CO_2_. For the cell treatment experiments, pZIF-8@Bai and control compounds were freshly diluted to the desired concentrations in complete DMEM just before use.

### 2.3 Simulation of in vitro ischemia with OGD/R and assessment of cellular activity

An *in vitro* model of RIRI was simulated using R28 cells. Oxygen-glucose deprivation was conducted for 6 h under hypoxic conditions (1% O_2_, 5% CO_2_) in glucose-free medium. Reoxygenation was then carried out for 2 h under normoxic conditions (21% O_2_, 5% CO_2_) in complete medium to complete the OGD/R cycle. All experimental groups, unless otherwise specified as “Control”, underwent oxygen-glucose deprivation/reoxygenation (OGD/R) prior to treatment. Cell viability and cytotoxicity were assessed using CCK-8 and live/dead assays, respectively. To determine the optimal concentration of pZIF-8@Bai, R28 cells were seeded in 96-well plates (5000 cells/well) for 12 h, then treated with ZIF-8, pZIF-8, and pZIF-8@Bai (5–35 μg/mL) for 24 h. Following OGD/R induction, viability was measured by CCK-8 assay per manufacturer's instructions, with calculations based on OD-value at 450 nm. For cytotoxicity evaluation, drug-treated and OGD/R-exposed R28 cells were stained with Calcein-AM/PI to distinguish viable from non-viable populations.

### 2.4 Detection of intracellular ROS

Following a 24-h treatment with the respective NPs (ZIF-8, pZIF-8, or pZIF-8@Bai; 25 μg/mL) and subsequent OGD/R exposure, intracellular ROS levels in R28 cells were analyzed by Leica fluorescence microscopy using DCFH-DA staining (1:1000, Beyotime, China). In parallel, MDA levels, SOD activity, catalase levels and ATP levels under identical treatment conditions were quantified using commercial assay kits (Beyotime, China). GSH levels under identical treatment conditions were quantified using commercial assay kits (Solarbio, China).

### 2.5 Detection of JC-1

Mitochondrial membrane potential (ΔΨm) was quantitatively assessed in R28 cells utilizing JC-1 fluorochrome (Beyotime, China) following a 24 h NPs (ZIF-8, pZIF-8, or pZIF-8@Bai; 25 μg/mL) pretreatment period and subsequent induction of OGD/R. Cellular staining was performed with JC-1 working solution (20 min incubation), followed by sequential washing with assay buffer and resuspension in DMEM for immediate Leica fluorescence microscopy analysis.

### 2.6 Flow cytometric analysis

Following indicated treatments, R28 cells were collected, washed with PBS, and resuspended in 1× binding buffer. Cells were then stained with Annexin V-FITC and propidium iodide (PI) according to the manufacturer's instructions (Dojindo, Japan). After incubation in the dark at room temperature for 15 min, the samples were immediately analyzed by flow cytometry (BD Accuri™ C6) to distinguish viable, early apoptotic, and late apoptotic cell populations.

BV2 cells were plated in 6-well plates at a density of 5 × 10^5^ cells per well. Following a 24-h incubation with Bai, ZIF-8, pZIF-8, or pZIF-8@Bai, the cultures were exposed to Lipopolysaccharide (LPS, 500 ng/mL) for 12 h. Cells were then harvested, washed twice with DPBS, and centrifuged at 1000 rpm for 4 min. Subsequent fixation used 4% paraformaldehyde for 15 min, after which samples were washed again with DPBS and pelleted by centrifugation at 2000 rpm for 3 min. The fixed cells were incubated on ice with a cocktail of PE-conjugated anti-CD11b (1:150, BD Pharmingen, 557,397) and APC-conjugated anti-CD80 antibodies (1:200, BD Pharmingen, 560,016). Following three additional DPBS washes, the samples were analyzed. Data acquisition was performed on a flow cytometer (BD Accuri™ C6), and subsequent analysis utilized FlowJo software.

### 2.7 Immunocytochemistry

R28 cells and BV2 microglial cells were cultured in 24-well plates at a density of 1 × 10^5^ cells/well. R28 cells were treated as before and BV2 cells were first co-cultured with specified NPs for 24 h and then stimulated with LPS (500 ng mL^−1^; Sigma-Aldrich, USA) in serum-free medium to promote M1 polarization. For immunofluorescence staining, cells were fixed with 4% paraformaldehyde for 15 min at room temperature, followed by permeabilization with 0.5% Triton X-100. After blocking with 2% BSA for 30 min, the cells were incubated with primary antibodies diluted in blocking buffer at 4 °C overnight. Subsequently, samples were washed and incubated with Alexa Fluor 488 goat anti-rabbit IgG (H + L) antibody and Alexa Fluor 555 goat anti-mouse IgG (H + L) antibody (1:500, Invitrogen) for 1 h at room temperature, protected from light. Cell nuclei were counterstained with 4′,6-diamidino-2-phenylindole (DAPI, Beyotime, China), and images were acquired using a whole slide imaging system (3DHISTECH Pannoramic MIDI). Primary antibodies used were as follows: Phospho-NF-κB (1:1000, Cell Signaling Technology, 3033 S), NogoA (1:300, abcam, ab62024), TNF-α (1:250; Proteintech, 240702B7), IL-1β (1:200, ABclonal, A1112), IL-6 (1:250, Proteintech, 21865-1-AP), CD86 (1:500, abcam, ab119857), CD206 (1:500, abcam, ab64693).

### 2.8 Western blot

Total protein was extracted from cells using RIPA lysis buffer supplemented with protease and phosphatase inhibitors (Beyotime, China). Retinas were lysed in RIPA buffer supplemented with inhibitors and mechanically disrupted by vortexing with stainless steel or ceramic beads at 4 °C, followed by incubation and centrifugation to collect the supernatant. Protein concentration was determined with a BCA assay kit (Beyotime, China). Equal amounts of protein (20–50 μg) were separated by 10% SDS-PAGE and transferred onto PVDF membranes (Millipore, Germany). After blocking with 5% skim milk or BSA in TBST for 2 h at room temperature, the membranes were incubated with specific primary antibodies at 4 °C overnight. Following three washes with TBST, the membranes were probed with horseradish peroxidase (HRP)-conjugated secondary antibodies (anti-mouse or anti-rabbit, Beyotime, China) for 1 h at room temperature. Protein bands were visualized using an enhanced chemiluminescence (ECL) detection system and imaged with a ChemiDoc imaging system (Bio-Rad, USA). β-Actin was used as an internal loading control. Band intensities were quantified using ImageJ software (NIH, USA). Primary antibodies used were as follows: Phospho-NF-κB (1:1000, Cell Signaling Technology, 3033 S), NF-κB (1:1000, Cell Signaling Technology, #8242 S), Nogo-A (1:1000, abcam, ab62024), BCL-2 (1:1000, abcam, ab59348), BAX (1:1000, abcam, ab32503), β-Actin (1:20,000, Proteintech, 81115-1-RR), Phospho-PI3K (1:1000, Abmart, T40116), PI3K (1:1000, HUABIO, EM1701-62), Phospho-AKT (1:1000, HUABIO, ET1607-73), AKT (1:1000, Abmart, T40066).

### 2.9 Retinal ischemia/reperfusion injury (RIRI) induction and treatment

C57BL/6 mice, 6-8 weeks, weighing 18–23 g were obtained from the SJA Laboratory Animal Co., Ltd (Hunan, China). Mice were randomly allocated into experimental groups for subsequent interventions. RIRI was induced as previously described [28]. In brief, animals were anesthetized intraperitoneally with 0.3% pentobarbital sodium, and body temperature was maintained at 37 °C using a heating pad. Pupils were dilated with 1% tropicamide, and proparacaine hydrochloride eye drops were applied to the operative eye. A 32-gauge needle connected to a saline perfusion system was inserted into the anterior chamber. IOP was raised to approximately 90 mmHg by elevating the saline reservoir 160 cm above the eye level and maintained for 90 min. Mice exhibiting surgical complications such as aqueous humor leakage or lens injury were excluded from the study. One day after RIRI induction, mice were randomly allocated into five experimental groups: sham, saline, ZIF-8, pZIF-8, and pZIF-8@Bai. Each mouse received an intravitreal injection of the respective 1.5 μL formulation via a 33-gauge blunt needle after a limbal incision was made with a 30-gauge needle, followed by immediate application of ofloxacin eye ointment.

### 2.10 Visual function analysis

All full-field flash dark-adapted scotopic electroretinogram (ERG) recordings were performed according to the protocol established by the International Society for Clinical Electrophysiology of Vision (ISCEV) [29]. Subjects underwent a 12-h period of dark adaptation prior to electrophysiological recording. General anesthesia was induced via intraperitoneal administration of avertin (20 mg/kg), followed by pupillary dilation with topical application of 1% tropicamide. Core body temperature was maintained at 37 °C throughout the procedure using a feedback-regulated heating platform. Electrophysiological recordings were obtained through bilateral corneal contact with gold ring electrodes, with simultaneous subcutaneous placement of reference (mid-frontal region) and ground (tail) electrodes. The corneal interface was hydrated with a methylcellulose-based coupling gel. Retinal responses were elicited by a calibrated Ganzfeld stimulator delivering flash intensities ranging from 0.01 to 10.0 cd s m^−2^. Under dark-adapted conditions. Quantitative analysis included measurement of a-wave and b-wave amplitudes and implicit times.

FVEP recordings were conducted in accordance with established electrophysiological protocols [30]. Following anesthetic induction, subjects were secured in a stereotaxic apparatus for precise electrode placement. Subdermal needle electrodes were positioned at standardized anatomical locations: the occipital protuberance (active recording electrode), the bregmatic suture (reference electrode), and the caudal region (ground electrode). Visual stimulation was delivered through a Ganzfeld system using standardized parameters: luminous intensity of 10.0 cd s/m^2^ at 1.4 Hz frequency, with 100 successive repetitions per recording session. Quantitative analysis focused on the peak latencies of the primary (P1) and secondary (P2) positive waveform components in the evoked response. All ERG and FVEP signal acquisition and processing were performed using a RETI-Port electrophysiology system (Roland Consult, Germany).

### 2.11 Detection of apoptosis

Detection of retinal apoptosis was performed using a TdT-mediated dUTP nick-end labeling (TUNEL) assay kit (Servicebio, China) in strict accordance with the manufacturer's protocol. In brief, retinal sections were fixed, permeabilized, and incubated with the TUNEL reaction mixture containing TdT enzyme and fluorescein-labeled dUTP. After counterstaining of cell nuclei with DAPI, the sections were mounted and visualized under the whole slide imaging system (3DHISTECH Pannoramic MIDI).

### 2.12 Hematoxylin-eosin (HE) staining and immunohistochemistry

Seven days post-retinal ischemia reperfusion injury and therapeutic intervention, murine ocular globes were enucleated and processed for histological analysis through standard fixation, dehydration, and paraffin embedding protocols. Sagittal sections (6 μm thickness) were obtained along the vertical meridian traversing the optic disc, maintaining parallelism to the maximal ocular circumference. Following deparaffinization, representative retinal sections underwent hematoxylin and eosin (H&E) staining for structural evaluation.

For immunohistochemical detection of cleaved caspase-8, sequential sections were subjected to antigen retrieval in citrate buffer (pH 6.0) after deparaffinization and rehydration. Endogenous peroxidase activity was eliminated by methanol-based 3% H_2_O_2_ quenching (15 min). Non-specific binding sites were blocked with 6% normal donkey serum (1 h) prior to incubation with anti-cleaved caspase-8 primary antibody (1:500, Cell Signaling Technology, #8592S). Immunocomplexes were visualized using the Vectastain ABC Elite detection system (Vector Laboratories, USA) with appropriate chromogenic substrate development.

### 2.13 Immunofluorescence staining

Immunofluorescence staining of retinal sections and retinal flat mounts was performed as formerly reported [31]. Following the designated therapeutic regimen in the RIRI model, subjects were euthanized and ocular globes were enucleated for histological processing. Tissues underwent fixation in 4% paraformaldehyde at 4 °C for 1 h, followed by cryoprotection through graded sucrose immersion. Specimens were subsequently embedded in Tissue-Tek® O.C.T. compound and subjected to rapid vitrification at −80 °C. Coronal retinal sections (10 μm) were obtained using a cryostat maintained at −22 °C, mounted on charged adhesive slides, and preserved at −20 °C. For immunofluorescence analysis, cryosections were brought to ambient temperature and rehydrated. Antigen retrieval was performed through permeabilization with 0.5% Triton X-100 in PBS for 30 min. Non-specific epitopes were blocked with a solution containing 3% donkey serum albumin and 0.3% Triton X-100 in 0.01 M PBS for 1 h at room temperature. Sections were incubated with primary antibodies at 4 °C for 16 h, followed by three washes in PBS. Species-specific secondary antibodies conjugated with fluorochromes were applied for 2 h at room temperature. Nuclear counterstaining was performed with DAPI for 5 min. Fluorescence images were acquired using a whole slide imaging system (3DHISTECH Pannoramic MIDI).

Immunohistochemical analysis of retinal whole mounts was performed as follows [32]. Immediately following enucleation, murine ocular globes were placed in ice-cold PBS, and retinas were meticulously dissected and rinsed. Tissues were fixed in 4% paraformaldehyde for 45 min, followed by three 5-min washes in PBS. Retinas were then blocked in a solution containing 3% donkey serum albumin and 0.3% Triton X-100 in 0.01 M PBS for 1 h at room temperature. Incubations with a primary antibody against Brn3a (1:500, abcam, ab245230) and Alexa Fluor 568-Isolectin B4 were carried out overnight at 4 °C, respectively. After three washes with PBS, samples were incubated with secondary antibody. Imaging was performed using a laser scanning confocal microscope (Dragonfly 200, Andor), and RGCs were quantified using ImageJ software.

### 2.14 Target screening

Stress-related gene sets, including “GOBP_CELLULAR_RESPONSE_TO_STRESS,” “GOBP_REGULATION_OF_CELLULAR_RESPONSE_TO_STRESS,” and “GOBP_REGULATION_OF_RESPONSE_TO_STRESS,” were retrieved from the MSigDB database. GSVA (version 2.2.1) was used to calculate pathway enrichment scores, and differential analysis of enrichment scores was performed using limma (version 3.66.0). The significantly enriched pathways were then intersected with the top 30 differentially expressed genes to identify key stress-related genes. To further prioritize candidate targets, the differentially expressed proteins were uploaded to the STRING database (version 12.0) for protein-protein interaction network construction with a confidence score cutoff of 0.4. The network was subsequently imported into Cytoscape, and seven algorithms in the CytoHubba plugin (MCC, MNC, Degree, EPC, Stress, Radiality, and Closeness) were applied to identify hub proteins. The top 50 proteins ranked by each algorithm were intersected, and the resulting hub proteins were then intersected with the previously identified stress-related genes derived from the significantly enriched pathways to determine the final target gene.

### 2.15 Molecular docking

Three-dimensional structures of the target proteins were obtained from the RCSB PDB database (https://www.rcsb.org/), and the structure with the lowest Å-resolution value was selected as the receptor. The 3D structures of core metabolites were downloaded from the PubChem database. Receptor preprocessing was performed in PyMOL, including removal of water molecules, addition of hydrogen atoms, and deletion of ligands and ions. Ligand structures were prepared in AutoDockTools by adding hydrogens, defining rotatable bonds and the geometric center, and calculating charges to minimize non-specific interference. Molecular docking was conducted using AutoDock Vina, with the docking box adjusted to fully cover the receptor's binding region and the number of docking runs set to 10. For each ligand–receptor pair, the pose with the lowest predicted binding energy was selected for downstream analysis.

### 2.16 Safety evaluation

The biosafety profile of intravitreally administered NPs was systemically evaluated. Seven days post-injection, retinal function was assessed by ffERG. Orbital blood samples were collected for biochemical analysis of hepatic and renal function parameters. Ocular globes and major organs (heart, liver, spleen, lungs, and kidneys) were harvested, embedded in paraffin. Tissue sections (6 μm) were prepared and stained with H&E for histopathological examination of structural integrity in retinal and systemic tissues.

### 2.17 Statistical analysis

Data are expressed as mean ± standard deviation (SD) from at least three independent experiments. Statistical analyses were performed using GraphPad Prism 8.0.2. For comparisons between two groups, a two-tailed Student's t-test was used. Multiple group comparisons were analyzed by one-way ANOVA followed by Tukey's post hoc test. A p-value <0.05 was considered statistically significant, denoted as p < 0.05, p < 0.01, and p < 0.001. Heatmaps were generated based on Z-score normalization.

## 3 Results

### 3.1 Preparation and characterization of pZIF-8@Bai

**Fig. 1:**
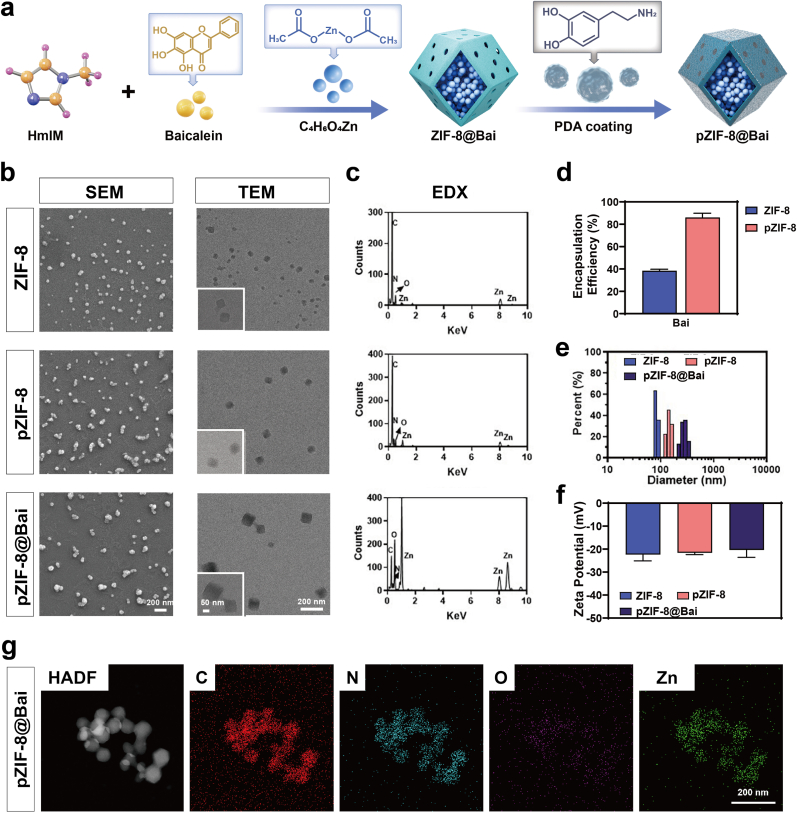
Characterization of ZIF-8, pZIF-8, pZIF-8@Bai. a) A schematic diagram outlining the synthesis of pZIF-8@Bai. b) SEM and TEM images of ZIF-8, pZIF-8, pZIF-8@Bai. Scale bar = 200 nm (Full TEM images). Scale bar = 50 nm (Partial TEM images). c) EDX spectra of ZIF-8, pZIF-8, pZIF-8@Bai samples. d) Encapsulation efficiency of ZIF-8 and pZIF-8, respectively. e) Particle size distribution of ZIF-8, pZIF-8, pZIF-8@Bai. f) Zeta potential of ZIF-8, pZIF-8, pZIF-8@Bai. g) Element mappings of pZIF-8@Bai (C, N, O, Zn). Scale bar = 200 nm. All results are presented as the mean ± SD.

In this study, Bai was successfully encapsulated into polydopamine-coated ZIF-8 via an in-situ synthesis approach to obtain pZIF-8@Bai NPs. The as-prepared ZIF-8 NPs were immersed in a basic dopamine (DA) solution, where DA underwent self-polymerization to form a polydopamine (PDA) functionalized surface, yielding pZIF-8. The study aimed to evaluate the effects of both in situ drug loading and PDA coating on ZIF-8 NPs (Fig. 1a). It is well established that the surface structure of nanodrug carriers significantly influences their sustained-release behavior, a property that is crucial for the treatment of retinal ischemia reperfusion injury. The materials were first characterized using scanning electron microscopy (SEM) and transmission electron microscopy (TEM). SEM images revealed that pZIF-8@Bai exhibits a larger particle size and a more spherical morphology with less distinct edges compared to ZIF-8 and pZIF-8 (Fig. 1b, left panels). TEM images confirmed that the in situ loading of Bai did not alter the ZIF-8 crystal morphology (Fig. 1b, right panels), and a thin gray translucent PDA layer was observed coating the surfaces of both pZIF-8 and pZIF-8@Bai (see insets for magnified views). Energy-dispersive X-ray (EDX) spectroscopy indicated that ZIF-8 and pZIF-8 exhibit similar spectral features with no significant differences in peak positions (Fig. 1c). X-ray photoelectron spectroscopy (XPS) survey spectra further demonstrated an increase in the intensity of oxygen-related peaks in pZIF-8, which is attributed to the successful coating of PDA on the ZIF-8 surface (Fig. S1a). The pZIF-8@Bai nanocarrier exhibits a sustained drug release profile, with Bai release reaching a plateau phase at approximately day 6 (Fig. S1b), under weakly acidic conditions (pH = 6.5), pZIF-8 released a greater amount of Bai (Fig. S2a), suggesting that Bai is encapsulated within ZIF-8 and also adsorbed onto the surface of pZIF-8 through non-covalent interactions such as hydrogen bonding. The drug encapsulation efficiency (EE) of Bai was subsequently evaluated. Owing to the presence of polyphenolic functional groups, the EE of Bai increased markedly from 38.43% in ZIF-8 to 86.13% in pZIF-8, thereby enhancing its ocular bioavailability (Fig. 1d). Dynamic light scattering (DLS) measurements showed minor differences in particle size between ZIF-8 (115.38 ± 24.20 nm) and pZIF-8 (165.1 ± 14.52 nm), while pZIF-8@Bai NPs displayed a more uniform size distribution (241.43 ± 17.8 nm), likely due to improved hydrophilicity (Fig. 1e). Zeta potential analysis was conducted to investigate the influence of PDA on the surface charge of the NPs. The results indicated surface potentials of −22.4 ± 2.26 mV for ZIF-8, –21.67 ± 0.60 mV for pZIF-8, and –20.4 ± 2.62 mV for pZIF-8@Bai (Fig. 1f). These values suggest that neither PDA modification nor Bai loading significantly alters the surface charge characteristics. The negative zeta potential contributes to excellent hydrophilicity and colloidal stability in aqueous dispersion. Elemental mapping of pZIF-8@Bai (Fig. 1g) confirmed the presence of C, N, O, and Zn as the primary constituents. Notably, the increased oxygen content originates from the abundant hydroxyl groups present in the PDA coating. This finding provides strong evidence that a uniform and continuous PDA layer was successfully formed on the surface of the ZIF-8 NPs. XRD analysis revealed that the as-prepared pZIF-8 displayed a characteristic phase structure highly consistent with standard ZIF-8 crystals (Fig. S3a), suggesting that PDA modification exerted no obvious influence on the structural stability and crystallinity of the ZIF-8 framework. As the FT-IR results shown in Fig. S4a, pure Bai exhibits a strong characteristic carbonyl (C=O) absorption peak of flavonoids at 1634 cm^−1^. ZIF-8 shows a strong characteristic peak of imidazole ring C=N stretching at 1580 cm^−1^, along with the C–N stretching vibration peak at 1145 cm^−1^, and the Zn–N stretching vibration peak at 421 cm^−1^. PDA displays characteristic peaks at 1653 cm^−1^ (aromatic ring C=C), 1545 cm^−1^ (phenolic hydroxyl bending), and 1288 cm^−1^ (C–O stretching vibration), respectively. The pZIF-8 composite material simultaneously retains the characteristic peaks of ZIF-8 at 1588, 1145, and 421 cm^−1^, as well as the characteristic absorptions of PDA, with no obvious new peaks generated, indicating that the two components are combined through physical mixing and weak interactions. In the pZIF-8@Bai system, in addition to the aforementioned characteristic peaks of PDA and ZIF-8, the carbonyl absorption peak of baicalein at 1631 cm^−1^ can still be observed, and no significant shifts in the positions of each characteristic peak are detected, confirming that baicalein has been successfully loaded into the pZIF-8 carrier while maintaining the structural integrity of the material framework.

### 3.2 Cytotoxicity, ROS scavenging ability, and anti-apoptosis of NPs in R28 cells subjected to OGD/R

**Fig. 2:**
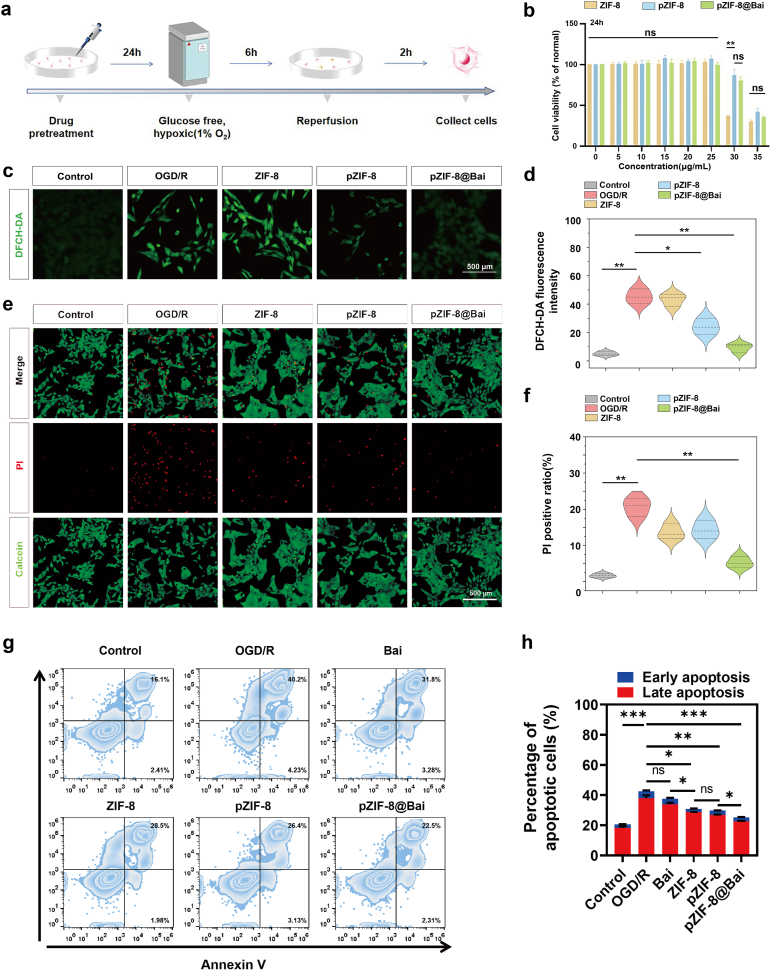
*In vitro* ROS scavenging and apoptotic inhibition of pZIF-8@Bai. a) Schematic illustration of the OGD/R model in R28 cells. b) Cell viability of R28 cells incubated with ZIF-8, pZIF-8, and pZIF-8@Bai at various concentrations (0−35 μg/mL) for 24 h (n = 5 per group). c) Representative fluorescence microscopy images of OGD/R-treated R28 cells stained with DCFH-DA after co-incubation with different drugs for 24 h (25 μg/mL). Scale bar = 50 μm. d) Quantitative analysis of ROS fluorescence intensity by Image J software. e) Representative fluorescence microscopy images of OGD/R-treated R28 cells were stained with Calcein-AM (green) and PI (red) after co-incubation with different drugs for 24 h (25 μg/mL). Scale bar = 500 μm. f) Quantitative analysis of PI positive ratio by Image J software. g) Representative flow cytometry plots of R28 cells stained with Annexin V and PI. Cells in the lower right quadrant (Annexin V^+^/PI^−^) represent early apoptotic cells, and cells in the upper right quadrant (Annexin V^+^/PI^+^) represent late apoptotic cells. h) Quantitative analysis of early apoptotic (blue bars) and late apoptotic (red bars) cells from the flow cytometry data. All results are presented as the mean ± SD (n = 3 per group). P values (b, d, f, and h) were determined by one-way ANOVA employing Tukey's post hoc test; all tests were two-sided; ns, not significant (p > 0.05); ∗p < 0.05, ∗∗p < 0.01, and ∗∗∗p < 0.001.

Before investigating the therapeutic effects of pZIF-8@Bai on oxygen-glucose deprivation/reoxygenation (OGD/R) in R28 cells (Fig. 2a), the biocompatibility and cytotoxicity profile of the nanocomposite were systematically assessed. A Cell Counting Kit-8 (CCK-8) assay revealed no significant adverse effects on R28 cell viability following 24-h exposure to varying concentrations of pZIF-8@Bai. However, a dose-dependent reduction in viability was observed at higher concentrations, which may be attributed to ZIF-8-induced mitochondrial dysfunction and ROS generation, as reported in previous studies (Fig. 2b) [33]. It is well-established that the overproduction of ROS, a key manifestation of oxidative stress, is instrumental in triggering retinal neurodegeneration and exacerbating RIRI [34]. Accordingly, therapeutic interventions designed to mitigate ROS accumulation hold significant promise. The antioxidative capacity of pZIF-8@Bai was further examined by measuring malondialdehyde (MDA) levels, glutathione (GSH) levels, superoxide dismutase, catalase levels, ATP levels and intracellular ROS using 2′,7′-Dichlorodihydrofluorescein Diacetate fluorescin diacetate (DCFH-DA). Among these NPs tested, pZIF-8@Bai (25 μg/mL) displayed the most pronounced cytoprotective effect under oxidative stress, decreasing MDA and increasing GSH, SOD, CAT, ATP levels (Fig. S5a–e). The result of DCFH-DA (Fig. 2c and d) showed lower ROS levels compared with the groups that were treated with ZIF-8 (25 μg/mL) and pZIF-8 (25 μg/mL). The free baicalein group exhibited a modest trend toward alleviating oxidative stress. In contrast, pZIF-8@Bai elicited a markedly more potent rescue, outperforming free drug across all metrics (Fig. S6a and b). Crucially, this redox recovery coincided with restored mitochondrial bioenergetics, evidenced by recovered ATP levels. We propose that by scavenging ROS and elevating the GSH levels, pZIF-8@Bai shields the electron transport chain from oxidative attack, thereby rescuing oxidative phosphorylation. This dual governance of oxidative stress and energy metabolism underpins reduced retinal cells apoptosis post-ischemia.

To verify the protective effect of pZIF-8@Bai against apoptosis, we used Calcein Acetoxy Methylester (Calcein-AM) and propidium iodide (PI) assay kit to count live/dead cells. The assay showed a significant increase in the survival rate of R28 cells subjected to OGD/R following treatment with pZIF-8@Bai, as compared to the OGD/R only group (Fig. 2e and f). Furthermore, under normal oxygen conditions, no notable alterations in Calcein-AM and PI staining were observed among ZIF-8, pZIF-8, and pZIF-8@Bai groups (Fig. S7a). These results imply that pZIF-8@Bai exerts a more pronounced anti-apoptotic effect in the context of OGD/R-induced injury relative to its nanoparticle counterparts. Compared to the control group, R28 cells subjected to OGD/R exhibited a significant increase in the percentage of apoptotic cells (both early and late apoptosis). Treatments with Bai, ZIF-8, pZIF-8, and pZIF-8@Bai all reduced the OGD/R-induced apoptosis to varying degrees. Notably, the pZIF-8@Bai treatment group showed the most pronounced protective effect, with the lowest levels of apoptotic cells among all treatment groups (Fig. 2g and h, Figure S8a).

### 3.3 PZIF-8@Bai alleviates oxidative damage and inflammation in R28 cells subjected to OGD/R

**Fig. 3:**
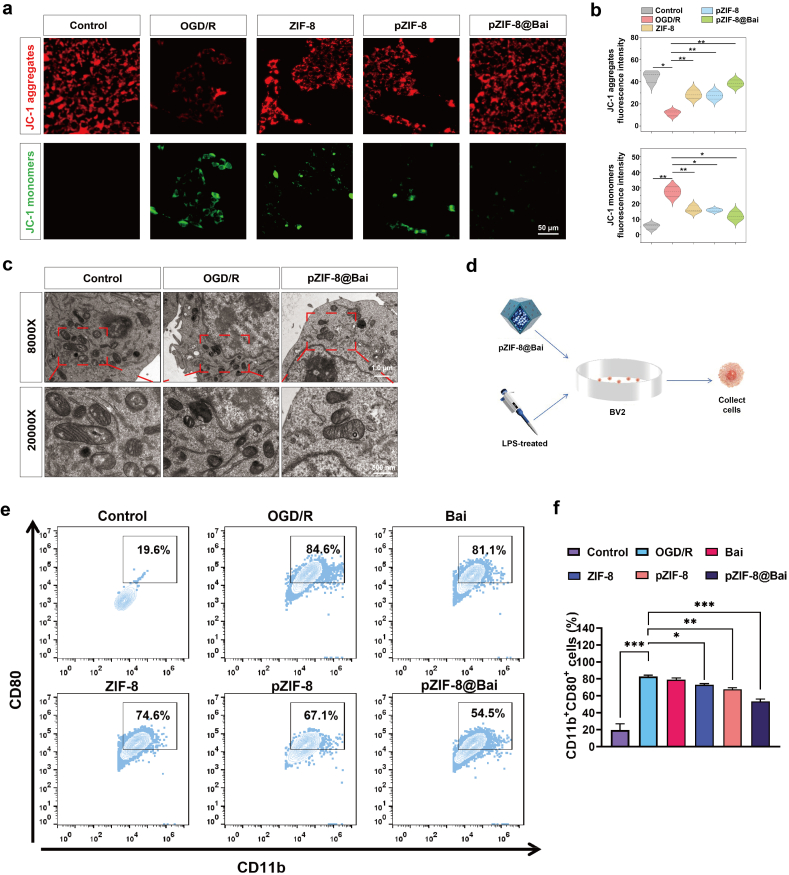
PZIF-8@Bai conferred mitochondrial protection in OGD/R-injured R28 cells and suppressed LPS-induced inflammation in BV2 cells. a) Representative fluorescence microscopy images of OGD/R-treated R28 cells stained with a JC-1 fluorescent probe after co-incubation with ZIF-8, pZIF-8, and pZIF-8@Bai for 24 h (25 μg/mL). Scale bar = 50 μm. b) Quantification of fluorescence intensity of JC-1 aggregates (red fluorescence) and JC-1 monomers (green fluorescence) by Image J software. c) TEM observation of mitochondrial membrane and cristae changes in R28 cells (mitochondria indicated by red boxes). d) A schematic illustrating the process of the LPS-induced inflammation in BV2 cells. e) Representative flow cytometric plots showing the expression levels of M1 macrophage markers (CD80^+^CD11b^+^) in BV2 cells following 24-h treatment with different experimental conditions. f) The percentages shown represent the proportion of CD80/CD11b double-positive cells. Data are presented as the mean ± SD (n = 3 per group). P values were determined by one-way ANOVA employing Tukey's post hoc test; all tests were two-sided; ns, not significant (p > 0.05); ∗p < 0.05, ∗∗p < 0.01, and ∗∗∗p < 0.001.

Mitochondria, fundamental organelles responsible for cellular energy metabolism and redox homeostasis, constitute a major endogenous source of reactive oxygen and nitrogen species (ROS/RNS) [35]. Overproduction or dysregulated production of these reactive species can precipitate oxidative stress, which plays a pivotal role in the pathogenesis of RIRI by disrupting mitochondrial integrity and function [36]. To assess mitochondrial health, the fluorescent probe JC-1 was widely utilized to monitor changes in mitochondrial membrane potential (ΔΨm). Under high ΔΨm conditions, JC-1 forms aggregates that emit red fluorescence, whereas depolarized mitochondria result in green fluorescent monomers. According to Fig. 3a, R28 cells subjected to OGD/R exhibited a marked decrease in mitochondrial membrane potential. Conversely, pZIF-8@Bai significantly restored the mitochondrial membrane potential and ameliorated mitochondrial damage (Fig. 3b), suggesting its protective role in maintaining mitochondrial integrity under ischemic stress. In contrast, free baicalein yielded a modest restoration of mitochondrial membrane potential, with its effect size falling short of that achieved by the pZIF-8@Bai (Fig. S9a and b).

TEM further revealed ultrastructural alterations in mitochondria, characterized by outer membrane disruption and cristae loss in R28 cells following OGD/R. These pathological features were substantially attenuated by pZIF-8@Bai treatment (Fig. 3c). Collectively, these findings indicate that pZIF-8@Bai confers cytoprotection by preserving mitochondrial integrity and suppressing apoptosis under OGD/R-induced injury.

Inflammation constitutes a pivotal pathological process in the pathogenesis and progression of RIRI [37]. RIRI triggers microglial hyperactivation and aberrant release of pro-inflammatory mediators, fostering a neurotoxic microenvironment that exacerbates retinal neuronal loss and degenerative remodeling [11]. To assess the anti-inflammatory efficacy of pZIF-8@Bai, an *in vitro* inflammatory model was established using BV2 microglial cells stimulated with lipopolysaccharide (LPS) (Fig. 3d). Immunofluorescence analysis of polarization markers revealed that LPS stimulation significantly increased CD86 expression (M1 phenotype), indicating a shift toward a pro-inflammatory state. In contrast, pZIF-8@Bai treatment markedly suppressed CD86 expression while promoting CD206 expression (M2 phenotype), indicating a shift toward an anti-inflammatory microglial polarization state (Fig. S10a and b). Flow cytometry analysis assessed the impact of various treatments on the expression of surface markers CD11b and CD80 in BV2 microglial cells. Compared to the untreated Control, stimulation with LPS significantly increased the population of CD11b^+^CD80^+^ cells, indicating successful M1 pro-inflammatory polarization. Treatment groups (Bai, ZIF-8, pZIF-8, pZIF-8@Bai) attenuated this LPS-induced effect to varying degrees. Among them, pZIF-8@Bai demonstrated the most potent inhibitory effect, achieving the lowest percentage of CD11b^+^CD80^+^ cells compared to the LPS group (Fig. 3e and f, Figure S11a). This suggests a superior synergistic anti-inflammatory efficacy for the pZIF-8@Bai formulation. To define the molecular basis of microglial regulation, we interrogated the PI3K/AKT axis. LPS stimulation triggered robust PI3K and AKT (Ser473) phosphorylation in BV2 cells, denoting pathway hyperactivation. pZIF-8@Bai potently suppressed these phosphorylation events, diminishing p-PI3K and p-AKT levels (Fig. S12a and b).

Furthermore, pZIF-8@Bai significantly attenuated the upregulation of key pro-inflammatory cytokines in R28 cells subjected to OGD/R, including IL-6, TNF-α, and IL-1β (Fig. S13a–f). These findings collectively demonstrate that pZIF-8@Bai exerts neuroprotective effects in the retinal inflammatory microenvironment by modulating microglial polarization and suppressing pro-inflammatory cytokine release. The PI3K/AKT pathway acts as a central signaling hub that processes inflammatory signals in microglia. The pZIF-8@Bai exerts its immunomodulatory effects by reducing the expression levels of p-PI3K and p-AKT. Under LPS stimulation, persistent activation of the PI3K/AKT pathway promotes NF-κB signaling and maintains M1 polarization. The pZIF-8@Bai suppresses this excessive pathway activity, which likely prevents p-NF-κB from entering the nucleus and reduces the expression of major pro-inflammatory factors.

### 3.4 PZIF-8@Bai attenuates retinal cells apoptosis in RIRI model

**Fig. 4:**
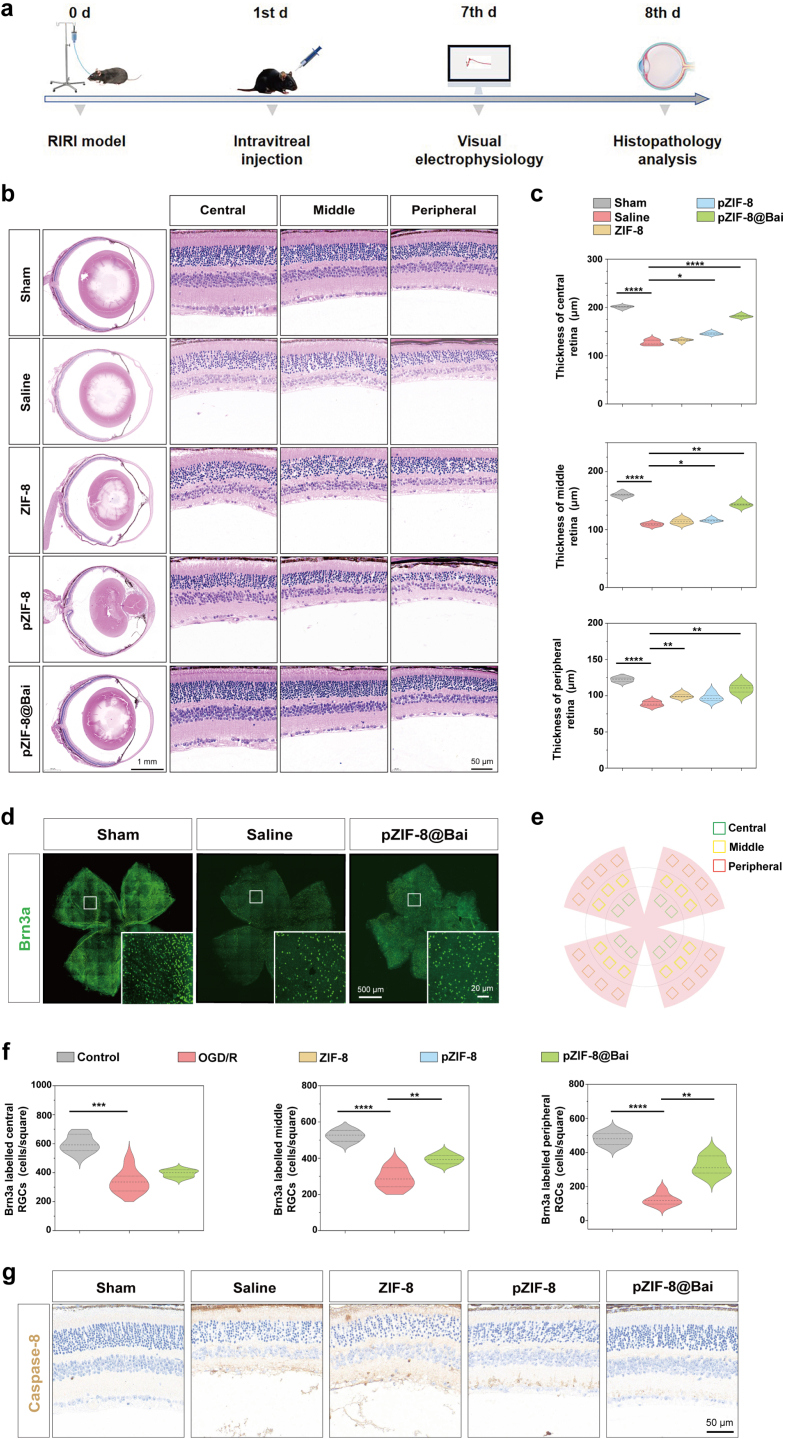
PZIF-8@Bai demonstrated marked efficacy in attenuating apoptosis in an *in vivo* RIR injury model. a) Schematic depicting the experimental paradigm and timeline for intravitreal pZIF-8@Bai therapy and subsequent validation in a rodent model of RIR injury. b) Representative H&E photomicrographs of retinal sections from the various experimental groups. Scale bar = 1 mm (Full retinal section). Scale bar = 50 μm (Partial retinal section). GCL, ganglion cell layer; IPL, inner plexiform layer; INL, inner nuclear layer; ONL, outer nuclear layer. c) Quantification of retinal full-thickness in the central, mid-peripheral, and peripheral regions from H&E staining across experimental groups. Data are presented as the mean ± SD (n = 5 mice per group). P values were determined by one-way ANOVA employing Tukey's post hoc test; all tests were two-sided; ns, not significant (p > 0.05); ∗p < 0.05, ∗∗p < 0.01, and ∗∗∗p < 0.001. d) Representative Brn3a (an RGC marker) stained retina flat mounts. Scale bar = 500 μm (Full retinal flat mounts). Scale bar = 20 μm (Partial retinal flat mounts). e) The diagram depicts the sampling scheme on a retinal section, illustrating how individual fields (analogous to puzzle pieces composing a larger montage) were acquired. As shown, the sampled areas in each quadrant are color-coded: green squares represent the central zone, yellow squares the mid-peripheral zone, and red squares the peripheral zone. Quantification of Brn3a-positive RGCs in a standardized area per quadrant from individual mice. f) Quantification of RGC number in central, middle-peripheral, and peripheral square areas are above shown. Data are presented as the mean ± SD (n ≥ 12 per group). P values were determined by one-way ANOVA employing Tukey's post hoc test; all tests were two-sided; ns, not significant (p > 0.05); ∗p < 0.05, ∗∗p < 0.01, and ∗∗∗p < 0.001. g) Immunohistochemical staining showing caspase-8 expression in retina sections from different groups 7 d after indicated intravitreal injection. Scale bar = 50 μm.

To systematically evaluate the therapeutic potential of pZIF-8@Bai in mitigating retinal neural injury, a well-established murine model of retinal ischemia reperfusion injury (RIRI) was employed. At 24 h post-RIRI induction, mice were randomly allocated into five experimental cohorts: (1) sham-operated, (2) saline-treated, (3) ZIF-8-treated, (4) pZIF-8-treated, and (5) pZIF-8@Bai-treated. A single intravitreal injection of the corresponding 1.5 μL formulation was administered to each group. Retinal tissues were harvested for histopathological examination on day 7 following the pharmacological intervention (Fig. 4a). This model reliably recapitulates the pathophysiological hallmarks of RIRI, allowing for a comprehensive assessment of neuroprotective interventions. Subsequent histopathological examination of retinal sections stained with hematoxylin and eosin (H&E) provided the initial structural evidence of damage and protection. The analysis revealed a pronounced reduction in total retinal thickness following RIRI induction compared to sham-operated controls, with prominent atrophy observed specifically in the central retinal region, which is particularly vulnerable to ischemic insult. Critically, treatment with pZIF-8@Bai demonstrated a significant protective effect, substantially mitigating this structural degeneration. The therapy significantly suppressed apoptosis in the RGC layer, inner nuclear layer (INL), and outer nuclear layer (ONL), thereby promoting a more compact and organized retinal cytoarchitecture that closely resembled that of healthy retinas (Fig. 4b and c). This suggested a broad, pan-retinal neuroprotective capacity.

To specifically quantify the survival of a critically important neuronal population, we performed quantification of Brn3a^+^ RGCs in retinal whole mounts. This analysis demonstrated a substantial increase in RGC density in the pZIF-8@Bai-treated group compared to saline controls, providing direct and conclusive evidence of its efficacy in preserving these key projection neurons, whose loss is a major cause of irreversible vision loss in RIRI and similar conditions (Fig. 4d–f). Furthermore, immunohistochemical analysis was conducted to identify the potential apoptotic pathways involved in neuroprotection of pZIF-8@Bai treatment. The results revealed a marked upregulation of the apoptotic initiator caspase-8 in RIRI-injured retinas, indicating the activation of the extrinsic apoptosis pathway in addition to previously implicated intrinsic pathways. This pathological upregulation was significantly attenuated following therapeutic intervention with pZIF-8@Bai. Notably, pZIF-8@Bai treatment resulted in the most pronounced suppression of caspase-8 expression among all experimental groups, underscoring its superior anti-apoptotic activity and its ability to modulate key initiators of cell death (Fig. 4g). Collectively, our results demonstrate the potent neuroprotective properties of pZIF-8@Bai against RIRI-induced structural damage and neuronal apoptosis.

### 3.5 PZIF-8@Bai alleviates retinal damage in RIRI mice model

**Fig. 5:**
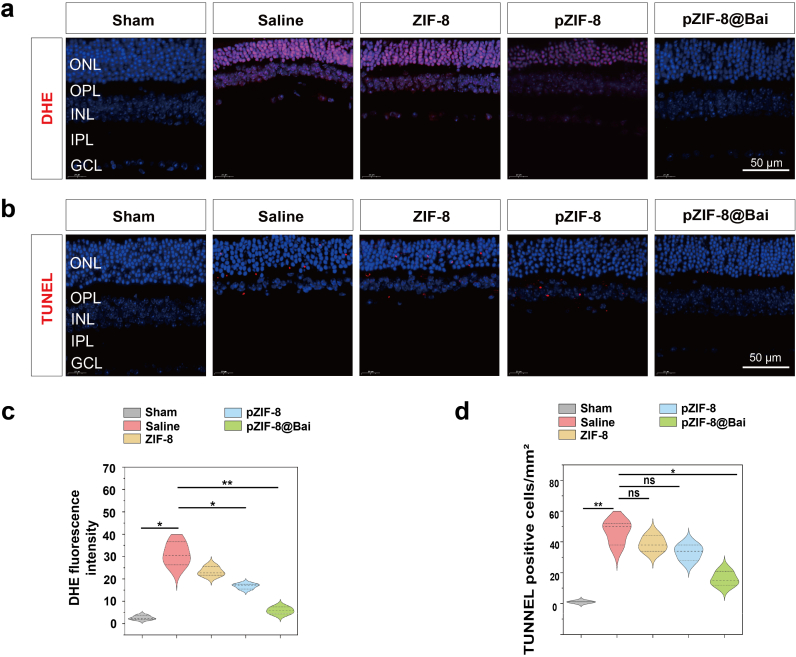
Etiological and histological analysis of the protective effects of pZIF-8@Bai. a) Representative fluorescence microscopy images of DHE staining showing retinal ROS levels in different groups. Scale bar = 50 μm. Immunostaining of TUNEL b), of retinal sections with the indicated treatment 7 d after RIR injury. Scale bar = 50 μm. Quantitative analysis of the fluorescence intensity of DHE c), TUNEL positive cells/mm^2^ d) in retinal sections was performed using ImageJ software. Data are presented as the mean ± SD (n = 5 mice per group). P values were determined by one-way ANOVA employing Tukey's post hoc test; all tests were two-sided; ns, not significant (p > 0.05); ∗p < 0.05, ∗∗p < 0.01, and ∗∗∗p < 0.001.

In the RIRI model, ischemic insult initiates a self-propagating cascade characterized by neuronal depolarization, pathological calcium influx, and energy depletion-induced oxidative stress. Subsequent reperfusion exacerbates tissue damage through reoxygenation-driven ROS generation [38], which amplifies inflammation [39], induces biomolecular damage, and culminates neuronal apoptosis [2,40]. As evidenced in Fig. 5a and c, pZIF-8@Bai treatment significantly attenuated the RIRI-induced retinal ROS accumulation and oxidative stress. The neuroprotective efficacy of pZIF-8@Bai was corroborated by a significant reduction in TUNEL-positive apoptotic cells following intravitreal administration (Fig. 5b and d). The consistency between these *in vivo* findings and corresponding *in vitro* data underscores the capacity of pZIF-8@Bai to mitigate associated apoptotic pathways.

### 3.6 PZIF-8@Bai preserves visual function in RIRI model mice

**Fig. 6:**
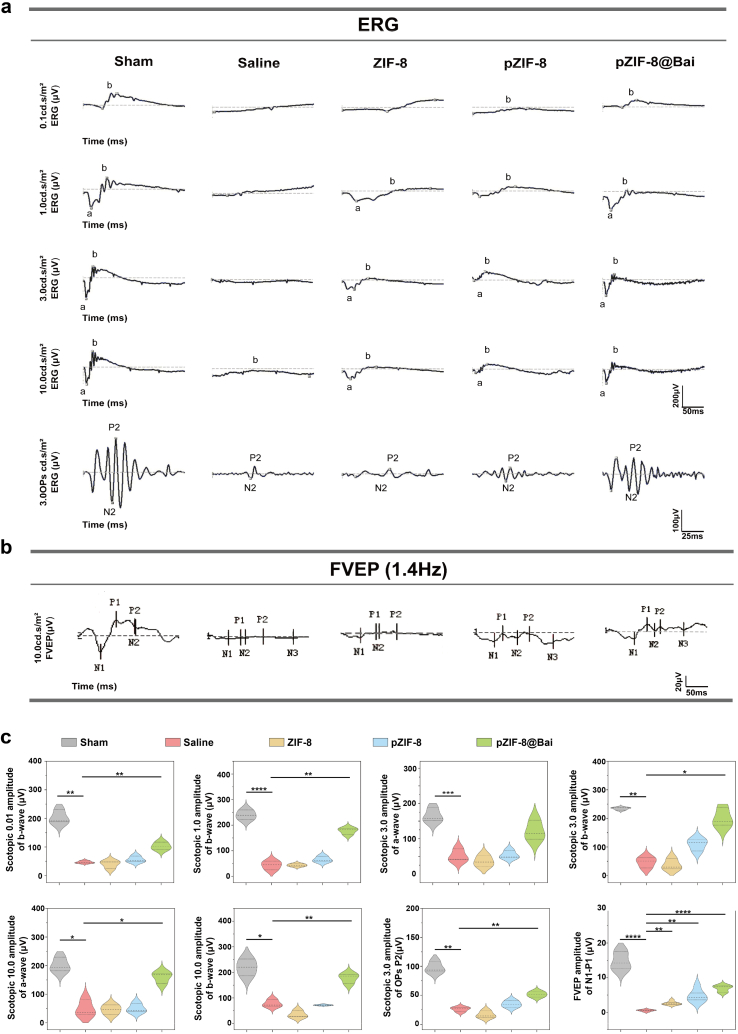
Retinal function was protected from RIR injury following intravitreal pZIF-8@Bai injection *in vivo*. a) Scotopic full-field electroretinography (ffERG) was performed across a range of flash intensities (0.1, 1.0, 3.0, and 10.0 cd s/m^2^) in mice after different treatments. b) Representative waveform of flash visual-evoked potentials (FVEPs) in mice after different treatments. c) The schematics illustrating amplitudes of the scotopic ffERG a-wave, b-wave, and oscillatory potentials in the groups at the different light intensity. Data are presented as the mean ± SD (n = 5 mice per group). P values were determined by one-way ANOVA employing Tukey's post hoc test; all tests were two-sided; ns, not significant (p > 0.05); ∗p < 0.05, ∗∗p < 0.01, and ∗∗∗p < 0.001.

To comprehensively evaluate the neuroprotective efficacy of pZIF-8@Bai on visual function after RIRI, we conducted a systematic assessment of the retinocortical pathway using full-field electroretinography (ffERG) and flash visual evoked potential (FVEP) recordings. The ffERG results under scotopic conditions provided a detailed functional map of the inner retina. Quantitative analysis revealed a severe attenuation of the a-wave, b-wave, and oscillatory potentials (OPs) in the saline-treated control group, indicating concurrent functional impairment in photoreceptors, bipolar cells, and amacrine cells, respectively. In stark contrast, treatment with pZIF-8@Bai prompted a stratified recovery pattern. Notably, the a-wave amplitudes, which reflect photoreceptor activity, exhibited a significantly greater restoration compared to the b-waves, which are associated with bipolar and Müller cells. This differential recovery suggests a preferential protective effect of pZIF-8@Bai on the outer retinal circuitry (Fig. 6a). To assess the functional integrity of the entire visual pathway beyond the retina, we performed FVEP recordings. The results demonstrated a significant enhancement of P1-wave amplitudes in the pZIF-8@Bai treated group compared to the saline controls. The preserved P1-wave, which originates from the primary visual cortex, indicates that the neural signals generated by the rescued retina are being transmitted more effectively through the optic nerve and central visual pathways (Fig. 6b and c). Therefore, the combined electrophysiological evidence—from the robust recovery of the photoreceptor-specific a-wave to the strengthened cortical response—logically demonstrates that pZIF-8@Bai treatment effectively preserves the functional integrity across the entire retinocortical pathway following RIRI.

### 3.7 Biosafety assessment of pZIF-8@Bai

To ascertain the intrinsic biocompatibility of pZIF-8@Bai under physiological conditions, the nanocomposite was administered via intravitreal injection to healthy C57BL/6 mice, with phosphate-buffered saline (PBS) serving as a vehicle control (Fig. S14a). A comprehensive histopathological and functional assessment of the retina integrity was conducted 7 days post-injection. Histological analysis revealed that the retinal architecture in pZIF-8@Bai-treated mice remained intact and well-organized, with retinal thickness comparable to that of the PBS-treated controls (Fig. S14b and c). Isolectin B4 staining of retinal whole mounts showed no significant differences in vascular morphology, including radial expansion, density, or branching complexity, between the pZIF-8@Bai and PBS groups (Fig. S14d). Functional integrity was further confirmed by ERG, indicating that the pZIF-8@Bai did not impair normal retinal electrophysiological function (Fig. S14e).

A rigorous evaluation of the systemic toxicological profile of pZIF-8@Bai constitutes an indispensable component of its preclinical safety assessment. To investigate the potential for off-target toxicity, a comprehensive analysis of hematological parameters and serum biochemical markers was conducted in a murine model 7 days post-intravitreal administration of pZIF-8@Bai. No statistically significant alterations relative to controls were observed across a range of hematological indices, hepatic transaminase levels, or renal function biomarkers (Fig. S14f). Subsequent histopathological evaluation of principal organ systems through hematoxylin and eosin (H&E) staining corroborated the structural integrity of tissues and revealed no acute pathological manifestations (Fig. S14g). The combined datasets provide strong evidence for a lack of acute systemic toxicity, thereby substantiating the favorable biosafety profile of pZIF-8@Bai.

### 3.8 Proteomic analysis of retinal tissue in RIRI mice treated with PZIF-8@Bai

**Fig. 7:**
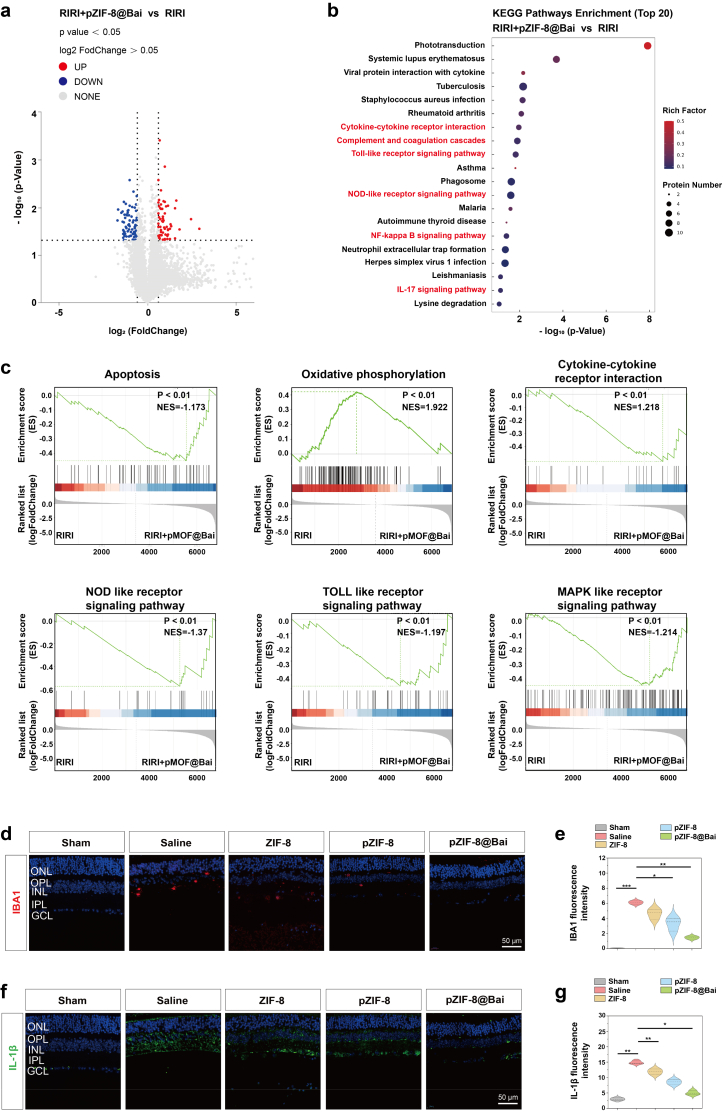
Proteomic analysis of the mouse retina in the RIRI and RIRI + pZIF-8@Bai groups. a) Volcano plot displaying DEPs in the retina from mice treated with RIRI + pZIF-8@Bai group versus RIRI group. b) KEGG enrichment analysis of selected DEPs (RIRI + pZIF-8@Bai vs. RIRI) in the mouse retina. c) GSEA of inflammatory, oxidative stress, and apoptotic signaling pathways derived from proteomic data. Immunostaining of IBA1 d), and IL-1β f) of retinal sections with the indicated treatment 7 d after RIR injury. Scale bar = 50 μm. Quantitative analysis of the fluorescence intensity of IBA1 e), and IL-1β g) in retinal sections was performed using ImageJ software. Data are presented as the mean ± SD (n = 5 mice per group). Statistical significance in a, b, c, e, and g was calculated via one-way ANOVA employing Tukey's post hoc test; all tests were two-sided.

To define the proteomic alterations underlying the neuroprotective effects of pZIF-8@Bai, retinal proteomes from RIRI mice treated with pZIF-8@Bai or saline were compared. A total of 8258 and 8210 distinct proteins were identified in the pZIF-8@Bai and saline groups, respectively, as shown by the statistical analysis and Venn diagram of the detected proteome (Fig. S15a and b). Of particular note, comparative proteomic profiling identified 77 DEPs (differentially expressed proteins) down-regulated and 63 up-regulated in the pZIF-8@Bai cohort relative to the saline group. The conserved order of magnitude in the total number of proteins identified between the two experimental conditions indicates that intravitreal pZIF-8@Bai administration elicits a targeted proteomic response without inducing widespread nonspecific protein depletion (Fig. S15c). A volcano plot and hierarchical clustering heat map illustrate the spectrum of statistically significant proteins, thereby delineating the pronounced disparities in the proteomic profiles between the pZIF-8@Bai and saline groups (Fig. 7a, Fig. S15d).

Next, functional profiling through GO (Gene Ontology) enrichment analyses, and KEGG (Kyoto Encyclopedia of Genes and Genomes) enrichment analyses were conducted to elucidate the functional implications of the identified proteomic alterations between the pZIF-8@Bai and Saline conditions. These analyses highlighted a significant overrepresentation of proteins implicated in apoptosis, antioxidant defense, and immune-related functions (Fig. 7b, Fig. S16a), providing a mechanistic basis for the neuroprotective efficacy of pZIF-8@Bai. Consistent with this, GSEA (Gene set enrichment analysis) results further demonstrated a significant enrichment in apoptosis, immune response, oxidative stress pathways in the control group, underscoring a concerted modulation of these pathways by pZIF-8@Bai treatment (Fig. 7c). Microglial activation and subsequent pro-inflammatory cytokines release represent pivotal drivers of the inflammatory cascade in RIRI pathology [41,42]. Immunofluorescence analysis of ionized calcium-binding adapter molecule 1 (IBA1, a microglial marker), and interleukin-1β (IL-1β) revealed a substantial upregulation in saline-treated retinas after RIRI, indicative of pronounced neuroinflammation. Conversely, pZIF-8@Bai administration resulted in significant attenuation of these inflammatory markers (Fig. 7d–g). The consistency between these *in vivo* findings and corresponding *in vitro* data underscores the capacity of pZIF-8@Bai to mitigate RIRI-induced neuroinflammation.

### 3.9 The pZIF-8@Bai suppresses RTN4/Nogo signalling pathways in vivo and in vitro

**Fig. 8:**
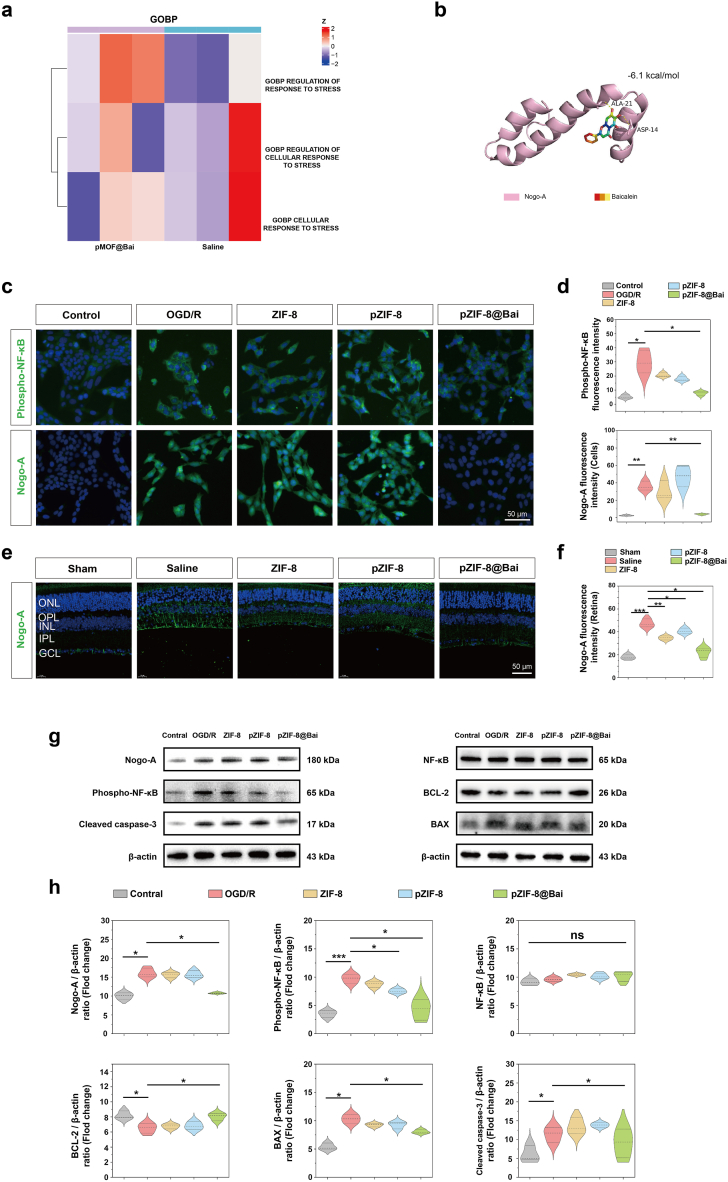
PZIF-8@Bai mediate their anti-inflammatory and anti-apoptotic effects through the Nogo signaling pathway. a) Limma analysis of the top 30 differentially expressed proteins and GO oxidative stress-related proteins. b) Molecular docking reveals specific binding of baicalein to RTN4. c) Representative fluorescence microscopy images of OGD/R-treated R28 cells stained with phospho-NF-κB and Nogo-A after co-incubation with ZIF-8, pZIF-8, and pZIF-8@Bai for 24 h (25 μg/mL). Scale bar = 50 μm. d) Quantification of fluorescence intensity of phospho-NF-κB and Nogo-A. e) Immunostaining of Nogo-A of retinal sections with the indicated treatment 7 d after RIR injury. Scale bar = 50 μm. f) Quantitative analysis of the fluorescence intensity of Nogo-A in retinal sections was performed. Protein expression g) and quantification h) of Nogo-A, NF-κB, phospho-NF-κB, BCL-2, BAX, and cleaved caspase-3 in R28 cells incubated with ZIF-8, pZIF-8, and pZIF-8@Bai (25 μg/mL) after OGD/R. All results are presented as the mean ± SD (n = 3 per group). P values (b, c, and e) were determined by one-way ANOVA employing Tukey's post hoc test; all tests were two-sided; ns, not significant (p > 0.05); ∗p < 0.05, ∗∗p < 0.01, and ∗∗∗p < 0.001.

Potential protein targets were prioritized using a GSVA analysis focused on a curated set of proteins, integrating two key criteria: the top 30 most significantly differentially expressed species and those mapped to the oxidative stress pathway in the GO database (Fig. 8a). This targeted screening nominated RTN4 and STAT2 as high-confidence candidates (Fig. S16b). PPI analysis showed that the differentially expressed proteins were uploaded to the STRING database (version 12.0) for protein-protein interaction network construction. At a confidence score cutoff of 0.4, the network contained 77 nodes and 476 edges (Fig. S17a). The network was subsequently imported into Cytoscape, and seven algorithms implemented in the CytoHubba plugin were used to identify hub proteins. Intersection of the top 50 proteins ranked by each algorithm yielded 39 hub proteins (Fig. S17b). Further intersection of these 39 hub proteins with the two previously identified differentially expressed proteins in GOBP_REGULATION_OF_RESPONSE_TO_STRESS identified a single overlapping target, RTN4 (Fig. S17c), further supporting RTN4 as the most relevant key target in our dataset.

Molecular docking simulations were subsequently employed, demonstrating that Bai, the principal bioactive compound in pZIF-8@Bai, possesses a specific and favorable binding affinity for the RTN4 protein (Fig. 8b). We have further refined the molecular docking analysis by performing detailed ligand-protein interaction profiling and rescoring the binding energy for the baicalein-RTN4 docking complex. Specifically, we used the Protein-Ligand Interaction Profiler (PLIP) to identify interaction types and interacting residues in the baicalein-RTN4 complexes. The analysis showed that baicalein interacts hydrophobically with Ile10, Ala21, and Ala28, while forming hydrogen bonds and polar contacts with Asp14, Arg20, and the backbone carbonyl group of Ala21 in RTN4 (Fig. S17d, Tables S1 and 2). To obtain a more accurate estimate of binding energy, we conducted Prime MM-GBSA calculations (Schrödinger), yielding an MMGBSA_dG_bind value of −18.21 kcal/mol, indicative of a relatively favorable binding interaction between baicalein and RTN4. Collectively, these computational results suggest that Ile10, Ala21, Ala28, Asp14, and Arg20 serve as key residues stabilizing the baicalein-RTN4 docking complex.

Reticulon proteins (RTNs) represent a class of membrane-associated proteins that orchestrate neuroanatomical plasticity and facilitate functional recovery following injury or pathology within the central nervous system (CNS) [43]. Among them, Nogo-A (RTN4A), a member of the reticulon 4 (RTN4) family, is recognized as a multifunctional protein whose varied neurobiological roles underlie its implication in numerous CNS pathologies [44]. Evidence from the literature links intracellular Nogo-A to the modulation of inflammatory responses and apoptotic cell death, mechanisms central to both neuroprotection and neurodegeneration [45]. Specifically, Nogo-A overexpression has been shown to drive microglial activation and the transcription of pro-inflammatory cytokines through the phospho-NF-κB p65 signaling pathway [46,47]. In the context of cerebral ischemia-reperfusion injury, Nogo-A is also associated with endoplasmic reticulum stress-induced neuronal apoptosis [48]. Concurrently, RTN4 modulates intracellular mitochondrial hypefusion and endoplasmic reticulum-mitochondria contact sites, thereby initiating the intrinsic apoptotic pathway [49,50]. Our findings demonstrate that Nogo-A (RTN4A) is significantly upregulated in response to retinal ischemia reperfusion injury, a phenomenon consistently observed in both *in vivo* and *in vitro* settings. This upregulation was closely associated with the concurrent activation of NF-κB-mediated pro-inflammatory signaling and endoplasmic reticulum stress-driven intrinsic apoptosis pathways. Crucially, therapeutic intervention with pZIF-8@Bai effectively attenuated Nogo-A expression, which in turn led to a suppression of NF-κB activation. This primary effect resulted in the marked suppression of the pro-inflammatory phenotype in activated microglia. In parallel, the treatment restored the critical balance between the anti-apoptotic protein BCL-2 and the pro-apoptotic protein BAX. This rebalancing directly inhibited the subsequent activation of caspase-3, thereby conferring significant protection against cellular apoptosis (Fig. 8c–h). We also found that Nogo-A was robustly upregulated in both the R28 cells subjected to OGD/R and the retinas of RIRI mice, establishing a conserved pathological response across *in vitro* and *in vivo* models. This upregulation was tightly coupled with NF-κB activation and dysregulation of the BCL-2/BAX ratio (Fig. S18a and b), implicating both inflammatory and intrinsic apoptotic pathways. In this study, Western blot analyses were performed with a sample size of n = 3 per group. While this aligns with common practice for initial mechanistic investigations and revealed clear biological trends, future validation with expanded sample sizes will enhance the statistical robustness of the conclusions.

Collectively, this mechanistic insight not only corroborates existing literature but further elucidates that the retinal protection offered by pZIF-8@Bai in RIRI is mechanistically rooted in the suppression of Nogo-A. This key action coordinately attenuates two major detrimental pathways: pro-inflammatory signaling cascades and intracellular apoptotic pathways.

## 4 Conclusion and discussion

The relentless progression of retinal neurodegenerative diseases, such as glaucoma and retinal vascular occlusions, is fundamentally driven by the complex pathological cascade of RIRI. This process initiates a devastating cycle where primary ischemic insult is dramatically amplified upon reperfusion, leading to excessive oxidative stress, dysregulated inflammation, and ultimately, the irreversible loss of retinal ganglion cells and visual function [51,52]. Contemporary therapeutic paradigms remain constrained by their singular targeting of isolated pathological pathways, failing to address the interconnected nature of RIRI mechanisms [53,54]. Furthermore, the presence of formidable ocular barriers—particularly the blood-retinal barrier—substantially compromises drug bioavailability, necessitating repetitive invasive administrations [55]. This study was therefore conceived to bridge these critical gaps by developing a novel, multifaceted therapeutic strategy that could simultaneously intervene at multiple points in the RIRI cascade while overcoming the inherent delivery challenges. We engineered a rationally designed nano-synergistic therapeutic platform, termed pZIF-8@Bai. This platform utilizes zeolitic imidazolate framework-8 (ZIF-8) as the carrier, where the interaction between polydopamine (PDA) and the flavonoid structure of baicalein (Bai) forms a highly efficient drug-loading platform. Both the ZIF-8 core and the polydopamine shell are loaded with Bai, a natural flavonoid derived from *Scutellaria baicalensis* that possesses well-documented antioxidant and anti-inflammatory properties, albeit with challenging pharmacokinetic profiles. This platform transforms the nanoparticle into a dynamic “biosponge”. This design confers multiple synergistic functions: PDA serves as the first line of defense by directly scavenging reactive oxygen species, while its bioadhesive properties prolong the retinal retention of Bai. The inflammatory microenvironment-triggered release of baicalein then provides an effective second line of defense, enabling spatiotemporally controlled multi-stage therapeutic intervention precisely at the site of pathological insult. In future work, we will further fine-tune the key parameters governing polydopamine self-polymerization and conduct in-depth investigations into the loading forms of baicalein onto pZIF as well as the underlying materials science mechanisms of their specific interactions.

The efficacy of this design was rigorously validated through comprehensive *in vitro* and *in vivo* investigation. In an R28 cellular model of OGD/R, pZIF-8@Bai demonstrated superior cytoprotection compared to its individual components. The nanocomposite significantly attenuated oxidative stress, as quantified by reductions in reactive ROS and MDA levels, and critically preserved mitochondrial bioenergetics through the restoration of mitochondrial membrane potential. TEM further confirmed the mitigation of ultrastructural mitochondrial damage. Beyond antioxidative effects, the nanocomposite exhibited profound immunomodulatory capabilities, effectively shifting microglial polarization from a pro-inflammatory M1 phenotype to an anti-inflammatory, reparative M2 phenotype, concurrently suppressing the release of key pro-inflammatory cytokines.

These promising *in vitro* findings were robustly translated into a mouse model of RIRI. A single intravitreal injection of pZIF-8@Bai conferred remarkable structural and functional neuroprotection. Histological analysis revealed significant preservation of retinal thickness and more orderly cellular architecture in the RGC layer. This was coupled with a stark reduction in TUNEL-positive apoptotic cells and caspase-8 expression, confirming potent inhibition of RIRI-induced RGC apoptosis. Immunofluorescence staining demonstrated significant attenuation of microglial activation and inflammatory mediators *in vivo*. Functional assessments showed that pZIF-8@Bai treatment led to significant recovery of a-wave, b-wave, and oscillatory potential amplitudes, indicating restored function across retinal neurons.

A seminal contribution of this work lies in the elucidation of the novel molecular mechanism underpinning the neuroprotective efficacy of pZIF-8@Bai. Unbiased proteomic profiling identified the reticulon protein Nogo-A (RTN4) as a critically upregulated mediator in RIRI. Subsequent investigation revealed that Nogo-A overexpression drives pathological progression through activation of the pro-inflammatory NF-κB signaling cascade and induction of endoplasmic reticulum stress-mediated intrinsic apoptosis in RGCs. Intervention with pZIF-8@Bai effectively suppressed Nogo-A expression, resulting in concomitant inhibition of NF-κB activation and rebalancing of the mitochondrial apoptotic pathway, favoring the anti-apoptotic BCL-2 over the pro-apoptotic BAX. The significance of our findings is amplified when contextualized within existing literature. While individual therapeutic properties of Bai and PDA have been reported, our study represents a paradigm shift by integrating them into a smart, responsive nanoplatform that overcomes their inherent limitations. More importantly, while other studies have focused on single aspects of RIRI pathology, our platform's demonstrated ability to concurrently quench ROS, modulate microglial polarization, suppress the Nogo-A/NF-κB axis, and inhibit RGC apoptosis represents a robust, multi-targeted approach uniquely suited to combat the vicious cycle of RIRI. Future applications should include evaluation of long-term efficacy in chronic disease models, detailed pharmacokinetic and biodistribution studies, and investigation of the nanomedicine's role in restoring retinal electrophysiological microenvironment in large animal models. Validation in higher-order animal models would further strengthen translational potential.

In conclusion, this study successfully demonstrates the development and validation of an innovative nano-synergistic therapy for RIRI. By combining ZIF-8 core, a therapeutic Bai payload, and a multifunctional PDA shell, we have created a platform that effectively breaks the vicious cycle of oxidative stress, RGC apoptosis, and neuroinflammation. Our work provides compelling evidence that pZIF-8@Bai preserves retinal structure and function through multi-mechanistic action, including novel suppression of the Nogo-A signaling pathway. The excellent biosafety profile further underscores its clinical potential for treating devastating retinal neurodegenerative diseases.

## Ethics approval and consent to participate

All animal experiments were approved by Institutional Animal Care and Use of Chongqing Medical University (Chongqing, China; permit number: IACUC-CQMU-2024-0938). All protocols of animal studies conformed to the Guide for the Institutional Ethical Review Committee of the School of Chongqing Medical University.

## Funding

This research was funded by Chongqing Talents Program Project (CSTC2022YCJH-BGZXM0163); Innovation and Development Joint Fund Project of Chongqing Natural Science Foundation (CSTB2024NSCQ-LZX0023); the National Natural Science Foundation of China (NO.82571207).

## CRediT authorship contribution statement

**Xin Liu:** Conceptualization, Data curation, Investigation, Methodology, Project administration, Writing – original draft. **Keke Huang:** Data curation, Investigation, Project administration, Validation, Writing – original draft, Writing – review & editing. **Zhiqing Lin:** Data curation, Investigation, Methodology, Project administration, Writing – original draft. **Min Tang:** Data curation, Investigation, Methodology, Validation, Writing – review & editing. **Qianyi Lin:** Data curation. **Wangdu Luo:** Data curation. **Jiaguo Yuan:** Data curation. **Junlong Yu:** Data curation. **Yujie Rao:** Data curation. **Peizeng Yang:** Resources. **Lin Xie:** Conceptualization, Data curation, Funding acquisition, Supervision, Writing – original draft, Writing – review & editing.

## Declaration of competing interest

The authors declare that they have no known competing financial interests or personal relationships that could have appeared to influence the work reported in this paper.

## Data Availability

Data will be made available on request.
