# Supplementary material for: An integrated nano-delivery platform via baicalein-loaded polydopamine-MOF for enhanced neuroprotection against retinal ischemia reperfusion injury
Source: Mater Today Bio. 2026 May 21;38:103239. doi: 10.1016/j.mtbio.2026.103239 (PMC13240834; doi:10.1016/j.mtbio.2026.103239)
Supplement: Multimedia component 1 [file mmc1.docx]

# Supplementary Information

**An Integrated Nano-Delivery Platform via Baicalein-Loaded Polydopamine-MOF for Enhanced Neuroprotection against Retinal Ischemia Reperfusion Injury​**

*Xin Liu ^a,b,1^, Keke Huang ^c,1^, Zhiqing Lin ^d,1^,* *Min Tang ^a,b^, Qianyi Lin ^a,b^,* *Wangdu Luo ^a^, Jiaguo Yuan ^a^, Junlong Yu ^a^, Yujie Rao ^a^, Peizeng Yang^b^, Lin Xie ^a, *^*

^a^ *Department of Ophthalmology, The Third Affiliated Hospital of Chongqing Medical University, Chongqing, 401120, China.*

^b^ *Chongqing Key Laboratory for the Prevention and Treatment of Major Blinding Eye Diseases，Chongqing* *400042, China.*

^c^ *Department of Ophthalmology, Affiliated Hospital of Southwest Jiaotong University, The Third People’s Hospital of Chengdu, Chengdu, 610031, China.*

^d^ *Department of Ophthalmology, West China School of Medicine, Sichuan University, Sichuan University affiliated Chengdu Second People's Hospital, Chengdu Second People's Hospital, Chengdu, 610031, China.*

^1^ *Authors contributed equally*

*^*^ Correspondence: E-mail: xielin@hospital.cqmu.edu.cn (Lin Xie)*

*Keywords:* Retinal ischemia reperfusion injury, Polydopamine, MOF, Retinal ganglion cells, Oxidative stress


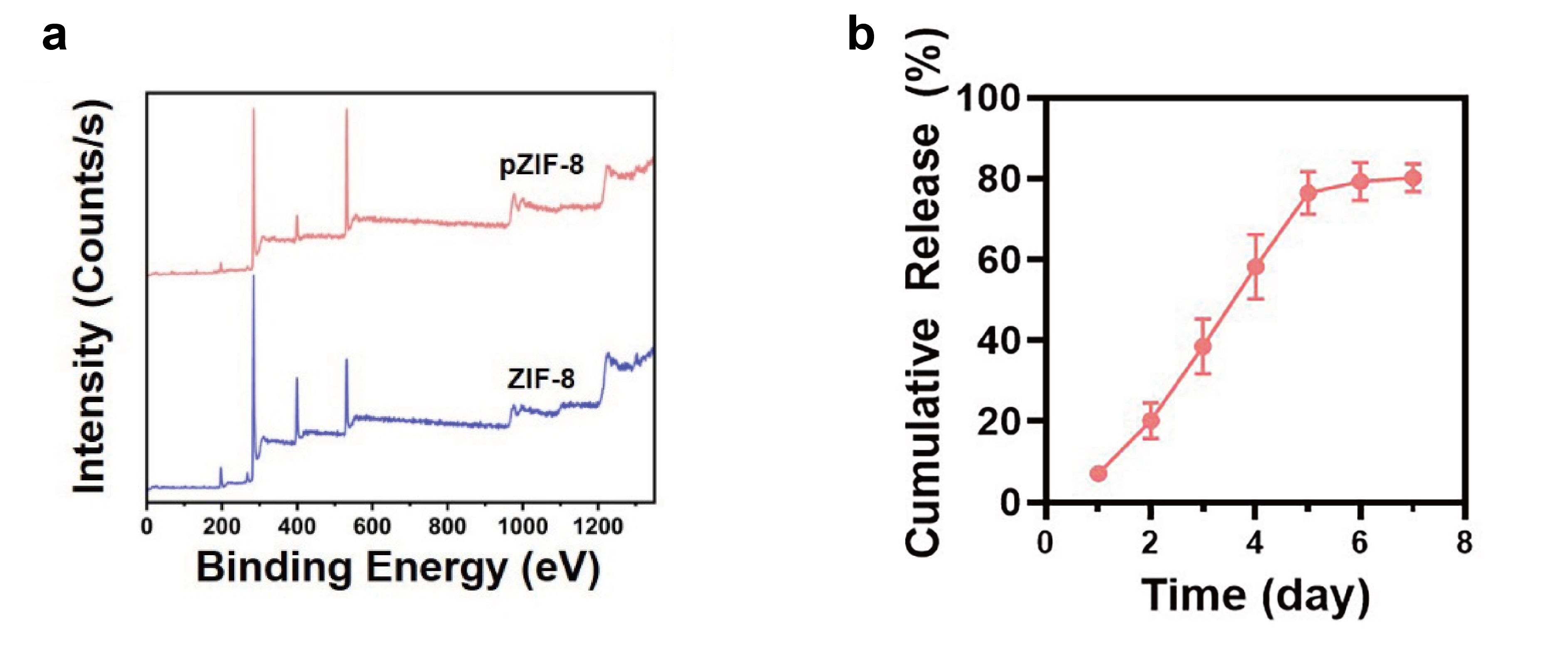


**Supplementary Figure 1. a)** XPS survey spectra of ZIF-8 and pZIF-8 particles. b) Cumulative release profiles of Bai from the pZIF-8 particles in PBS. Data are expressed as mean ± standard error of the mean (**p* < 0.05 and ***p* < 0.01; *n* = 3).


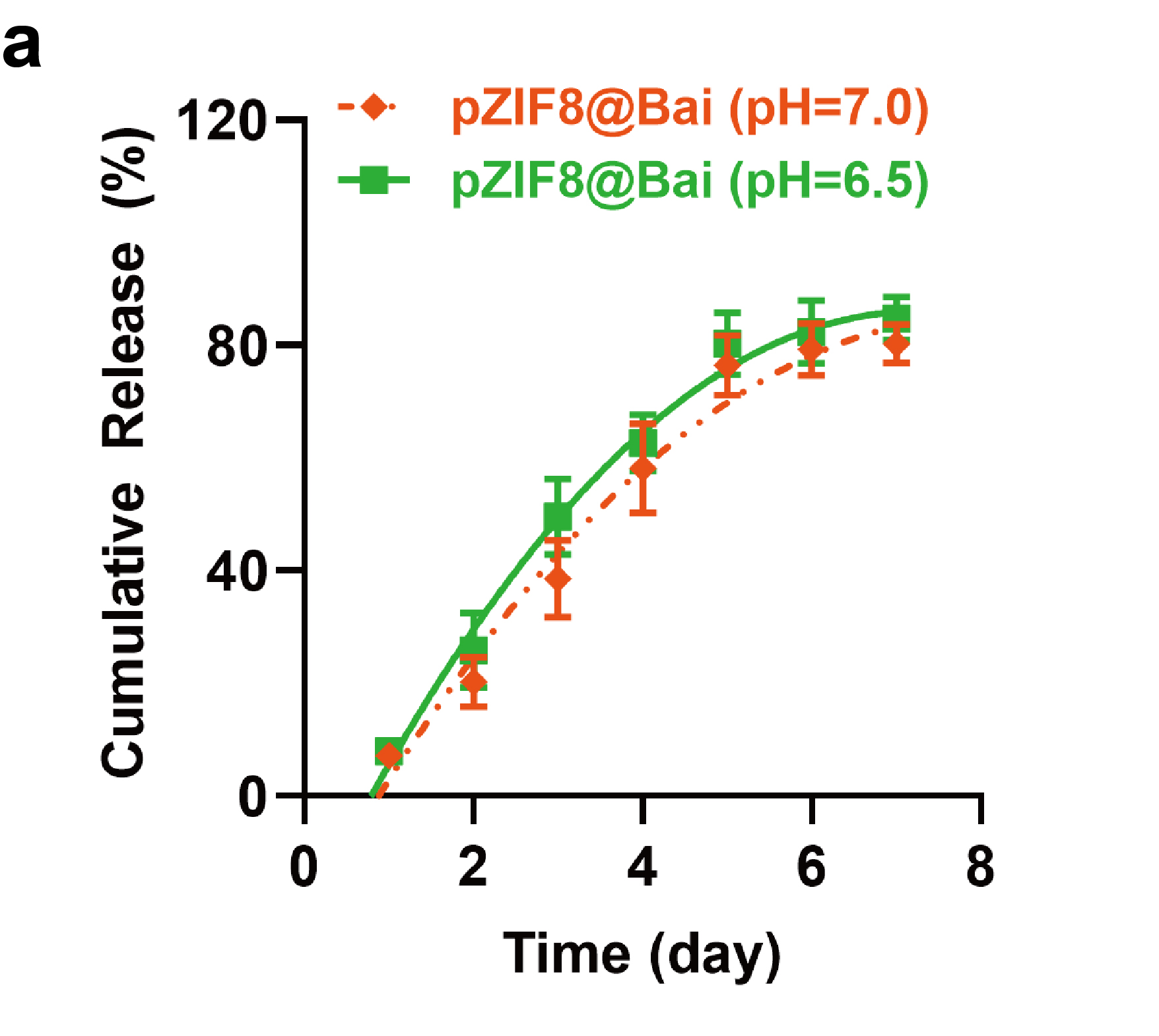


**Supplementary Figure 2. Loading and releasing curves of** Bai from pZIF-8@Bai**. a) A comparison of Bai delivery in various pH.**


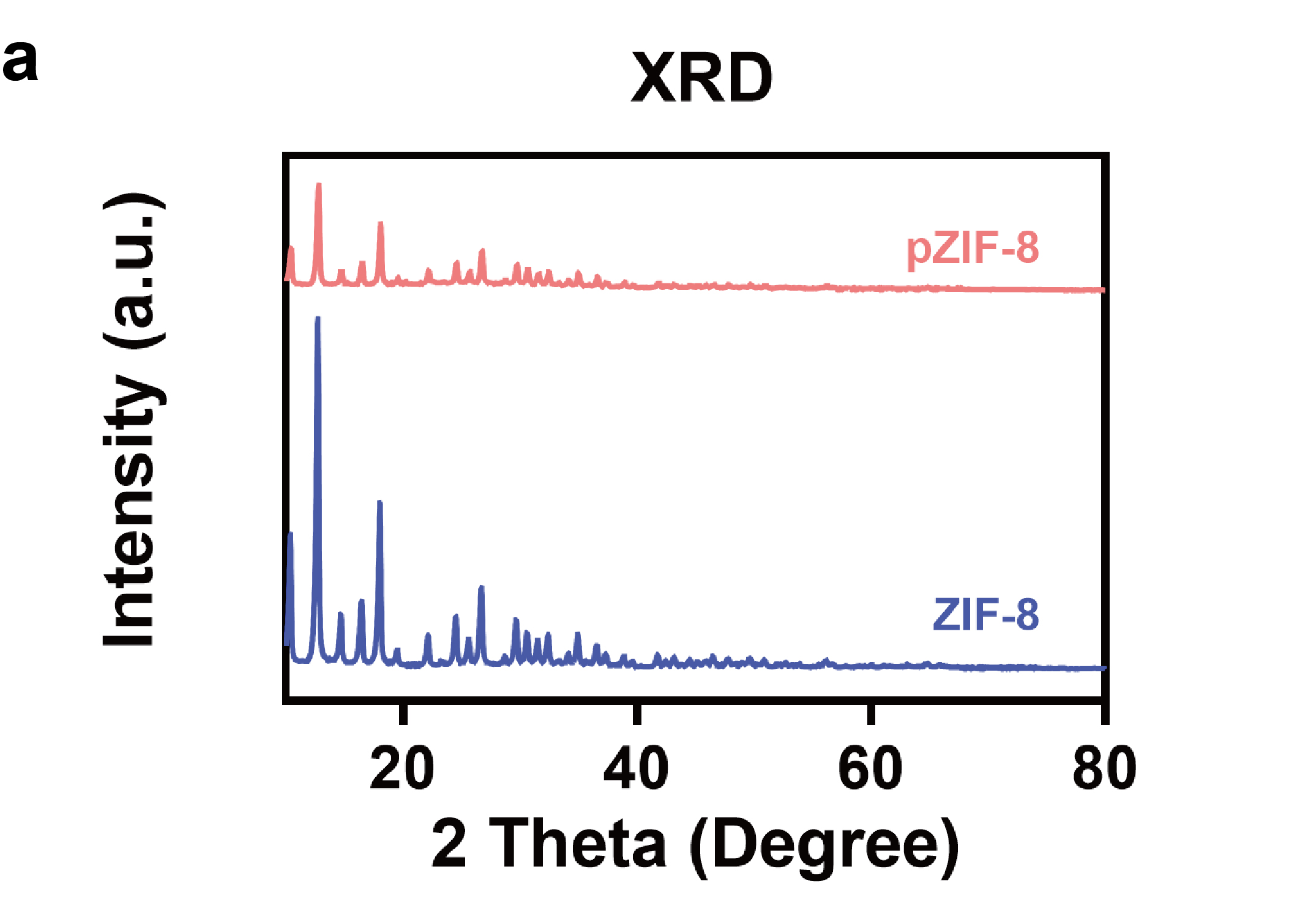


**Supplementary Figure 3. a) XRD phase analysis of pZIF-8** particles in PBS. Data are expressed as mean ± standard error of the mean (**p* < 0.05 and ***p* < 0.01; *n* = 3).

**
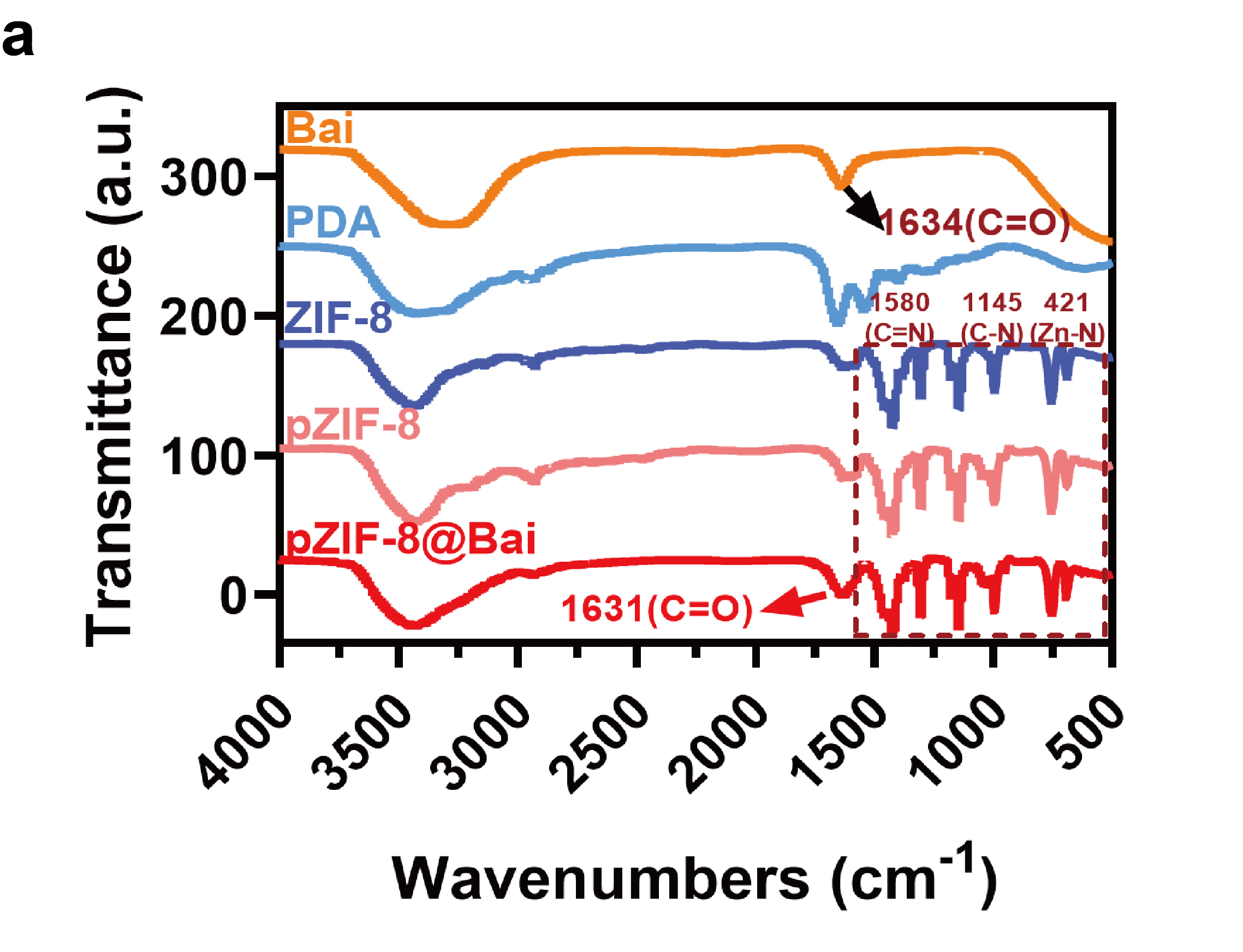
**

**Supplementary Figure 4. a) FTIR spectra of pZIF-8@Bai.** Data are expressed as mean ± standard error of the mean (**p* < 0.05 and ***p* < 0.01; *n* = 3).


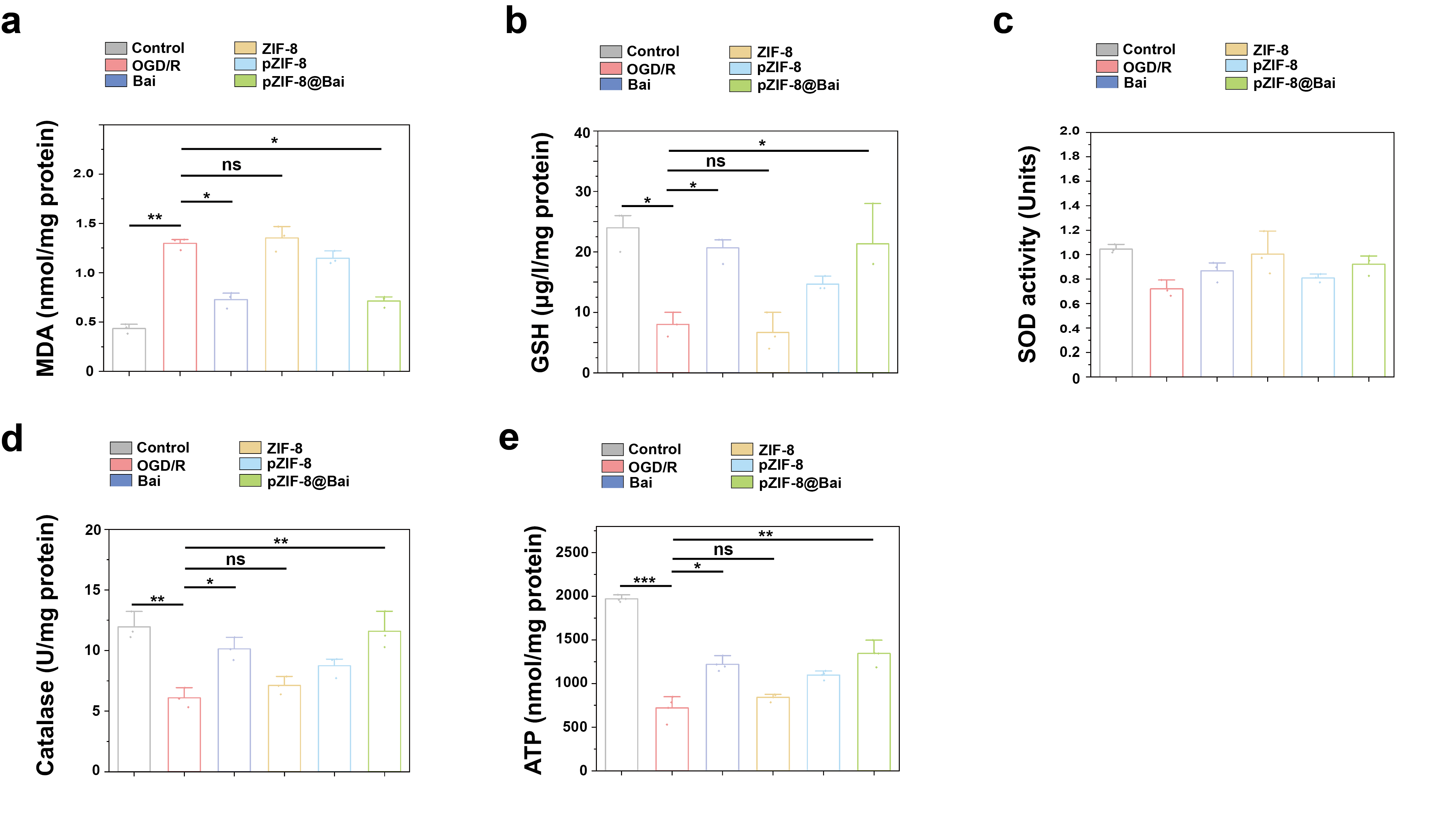


**Supplementary Figure 5. The MDA a), GSH b), SOD activity c), Catalase d) and ATP levels e) of R28 cells subjected to OGD/R with Bai, ZIF-8, pZIF-8, and pZIF-8@Bai for 24 h (n = 3 per group). All results are presented as the mean ± SD (n = 3 per group).** p values (a, b, d, and e) were determined by one-way ANOVA employing Tukey’s post hoc test; all tests were two-sided; ns, not significant (p > 0.05); * p < 0.05, ** p < 0.01, and *** p < 0.001.


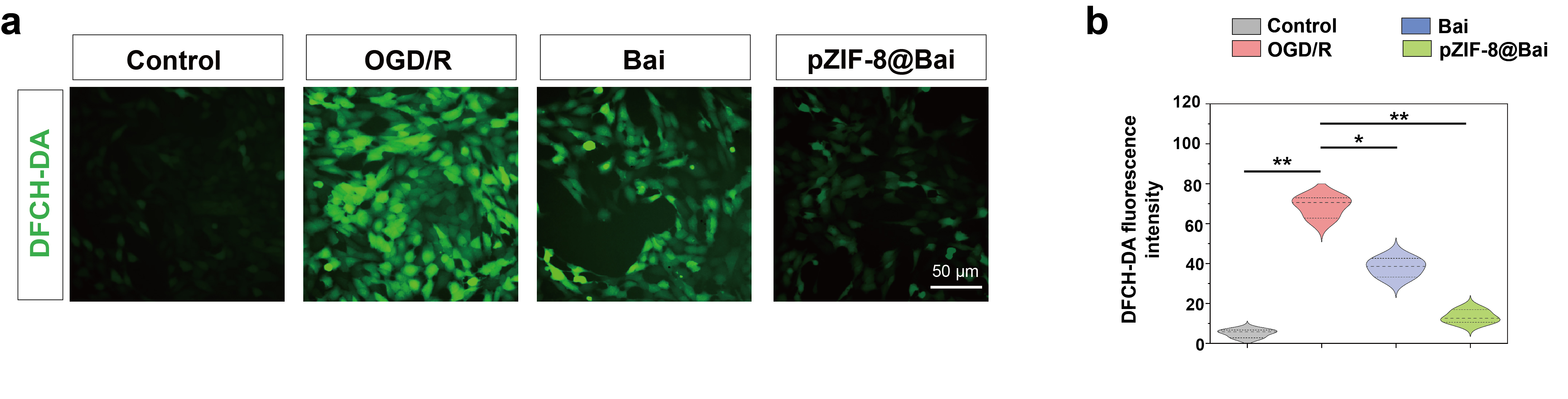


## ****Supplementary Figure 6.** a) Representative fluorescence microscopy images of OGD/R-treated R28 cells stained with DCFH-DA after co-incubation with Bai and pZIF-8@Bai for 24 h (25 μg/mL). Scale bar = 50 μm. b)** Quantitative analysis of **DCFH-DA** fluorescence intensity by Image J software**. Data are presented as the mean ± SD (n = 3 per group).** p values were determined by one-way ANOVA employing Tukey’s post hoc test; all tests were two-sided; ns, not significant (p > 0.05); * p < 0.05, ** p < 0.01, and *** p < 0.001.


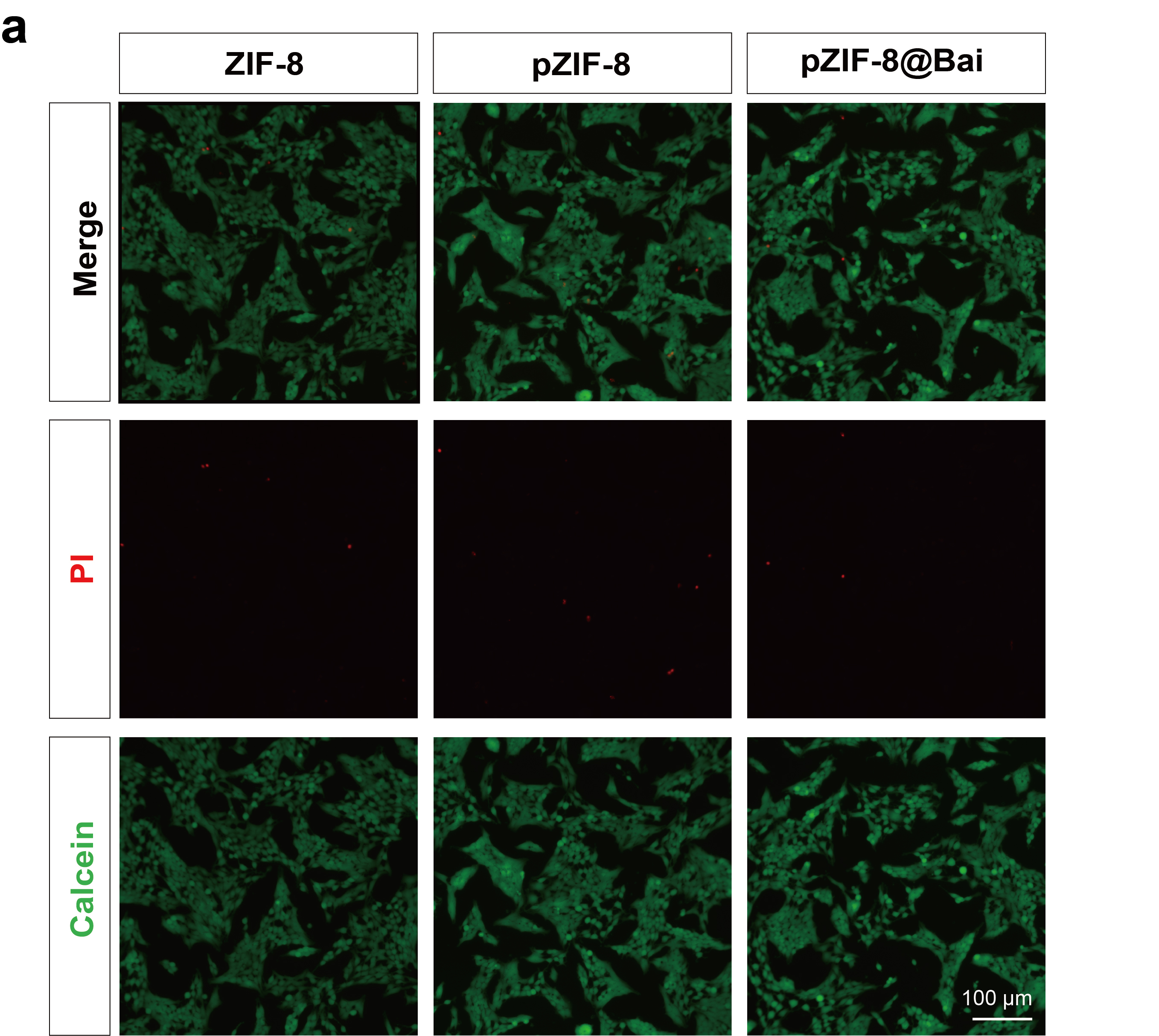


**Supplementary Figure 7. a) Representative fluorescence microscopy images of R28 cells** were stained with Calcein-AM and PI **after co-incubation with ZIF-8, pZIF-8, and pZIF-8@Bai for 24 h (25 μg/mL).Scale bar = 100 μm.**

**
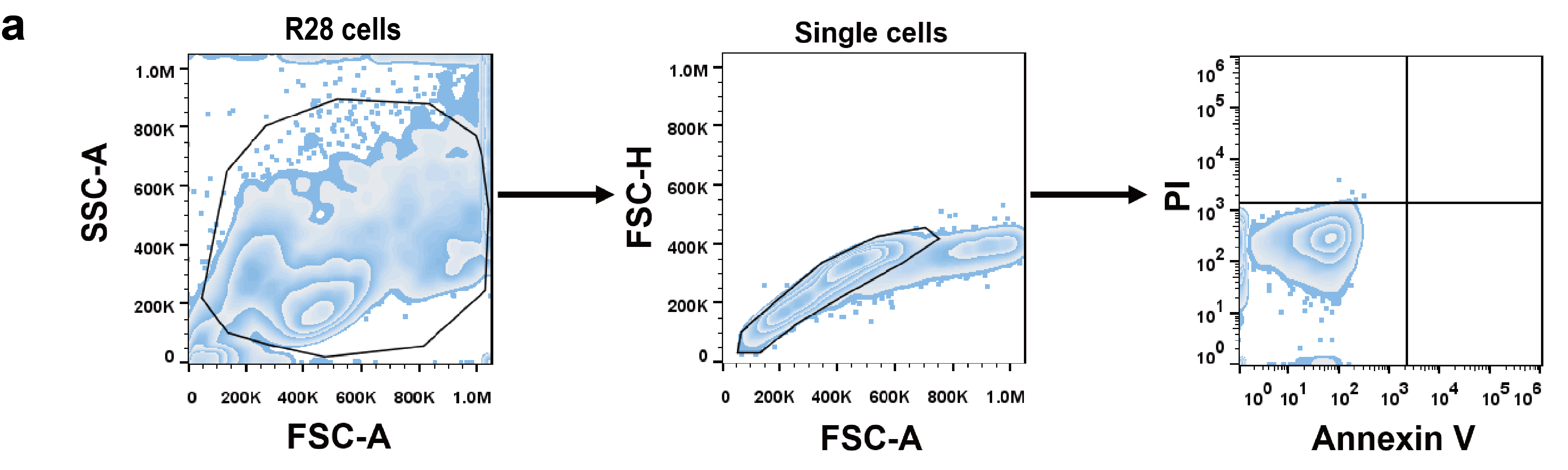
**

**Supplementary Figure 8. a)**  Flow cytometry gating strategy used for apoptosis in R28 cells.


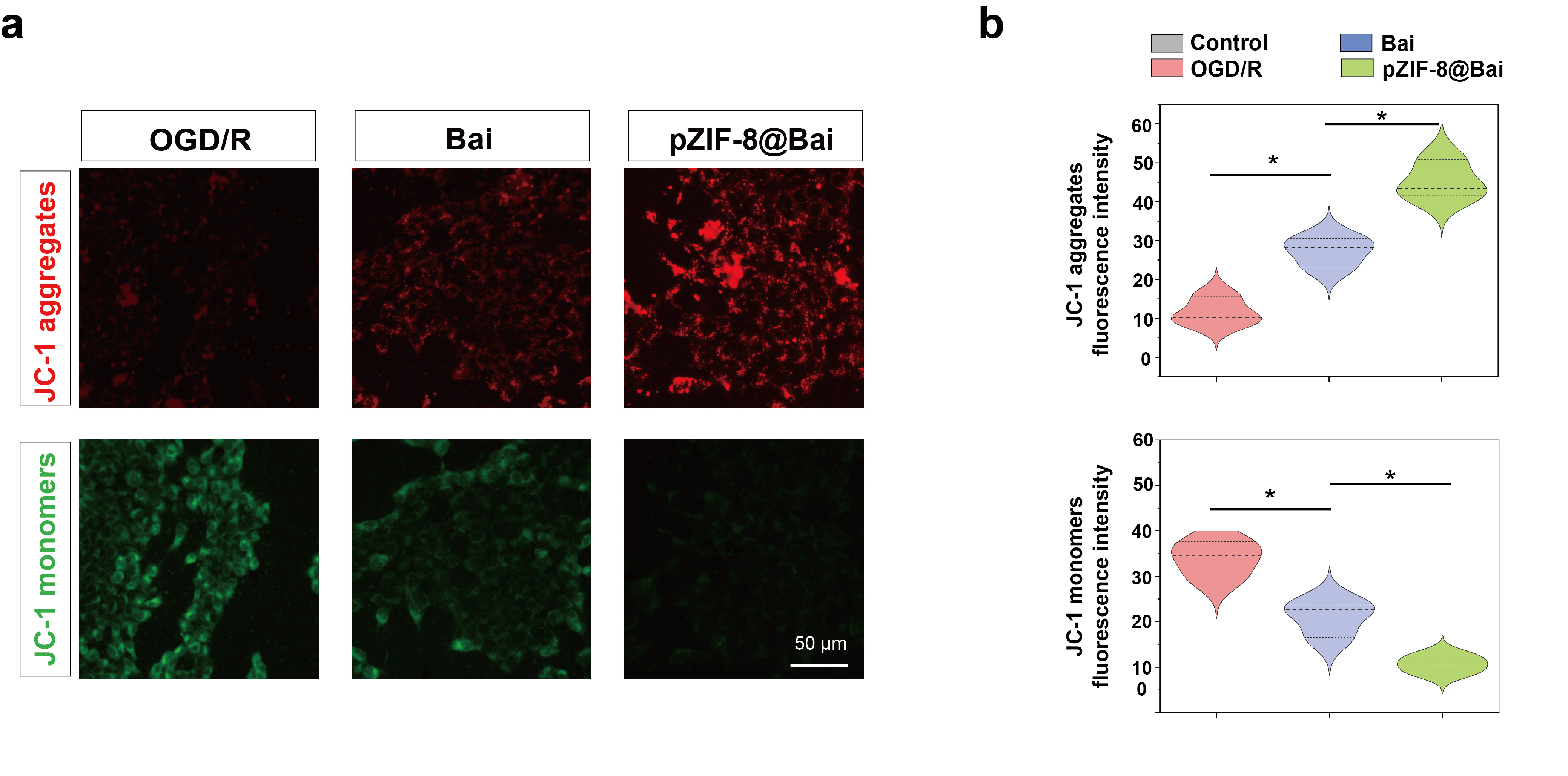


**Supplementary Figure 9. a) Representative fluorescence microscopy images** of **OGD/R-treated R28 cells** stained with a JC-1 fluorescent probe **after co-incubation with Bai and pZIF-8@Bai for 24 h (25 μg/mL)**. Scale bar = 50 **μ**m. b) Quantification of fluorescence intensity of JC-1 aggregates (red fluorescence) and JC-1 monomers (green fluorescence) by Image J software**. Data are presented as the mean ± SD (n = 3 per group).** p values were determined by one-way ANOVA employing Tukey’s post hoc test; all tests were two-sided; ns, not significant (p > 0.05); * p < 0.05, ** p < 0.01, and *** p < 0.001.


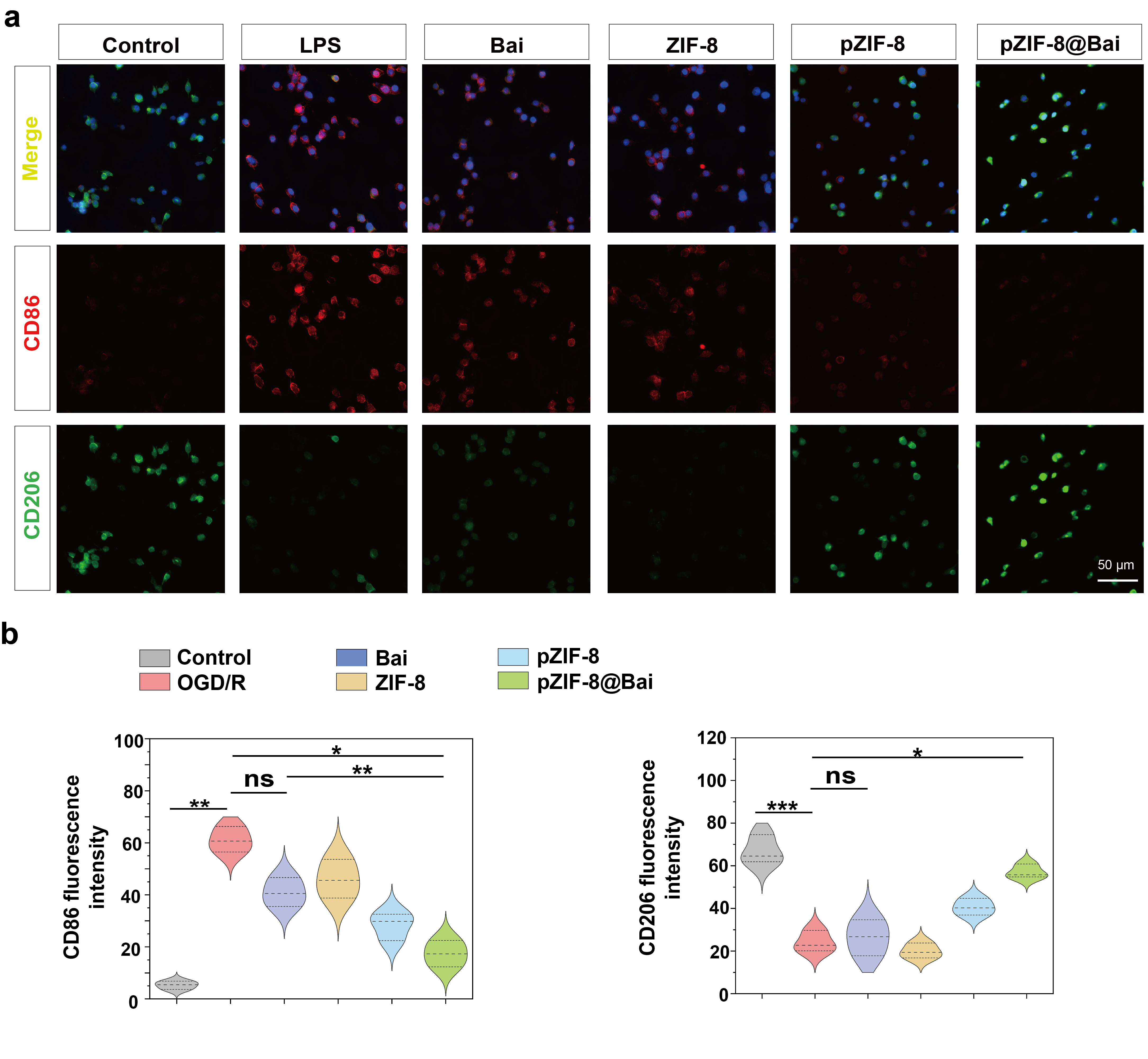


**Supplementary Figure 10. a) Representative fluorescence microscopy images** of **LPS-treated BV2 cells** stained with a CD86 (red) and CD206 (green) **after co-incubation with Bai, ZIF-8, pZIF-8, and pZIF-8@Bai for 24 h (25 μg/mL)**. Scale bar = 50 **μ**m. b) Quantitative analysis fluorescence intensity of CD86 and CD206 by Image J software**. Data are presented as the mean ± SD (n = 3 per group).** p values were determined by one-way ANOVA employing Tukey’s post hoc test; all tests were two-sided; ns, not significant (p > 0.05); * p < 0.05, ** p < 0.01, and *** p < 0.001.


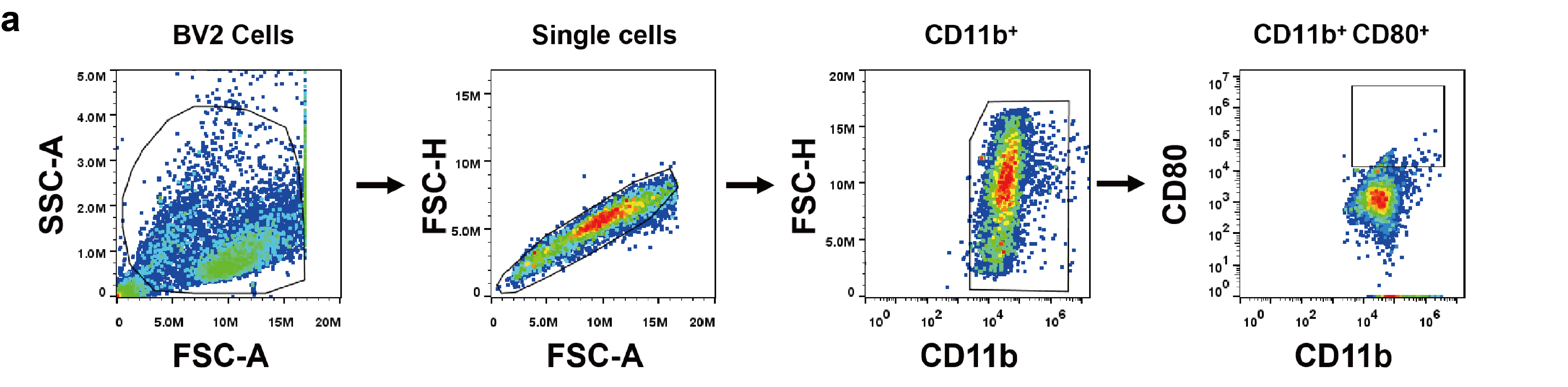


**Supplementary Figure 11. a)** Flow cytometry gating strategy used for identifying M1 macrophage subsets. BV2 cells were first gated to exclude cell aggregates and isolate single-cell populations. M1 macrophages were subsequently identified and quantified as CD11b⁺CD80⁺ double-positive cells.


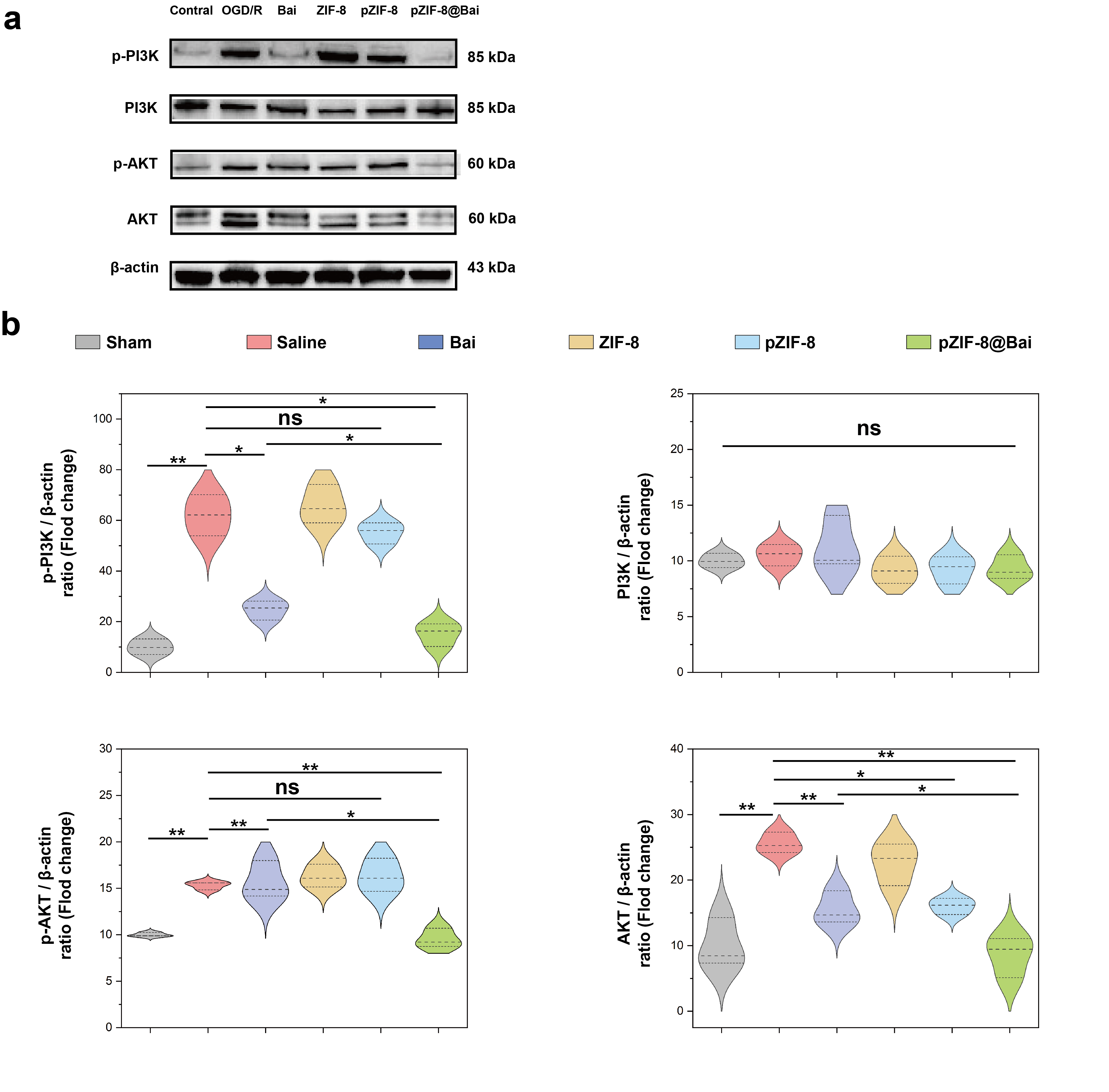


**Supplementary Figure 12.** Protein expression a) and quantification b) of phospho-PI3K, PI3K, phospho-AKT and AKT in BV2 cells **incubated with Bai， ZIF-8, pZIF-8, and pZIF-8@Bai (25 μg/mL)** after LPS-pretreated. All results are presented **as the mean ± SD (n = 3 per group).** p values were determined by one-way ANOVA employing Tukey’s post hoc test; all tests were two-sided; ns, not significant (p > 0.05); * p < 0.05, ** p < 0.01, and *** p < 0.001.


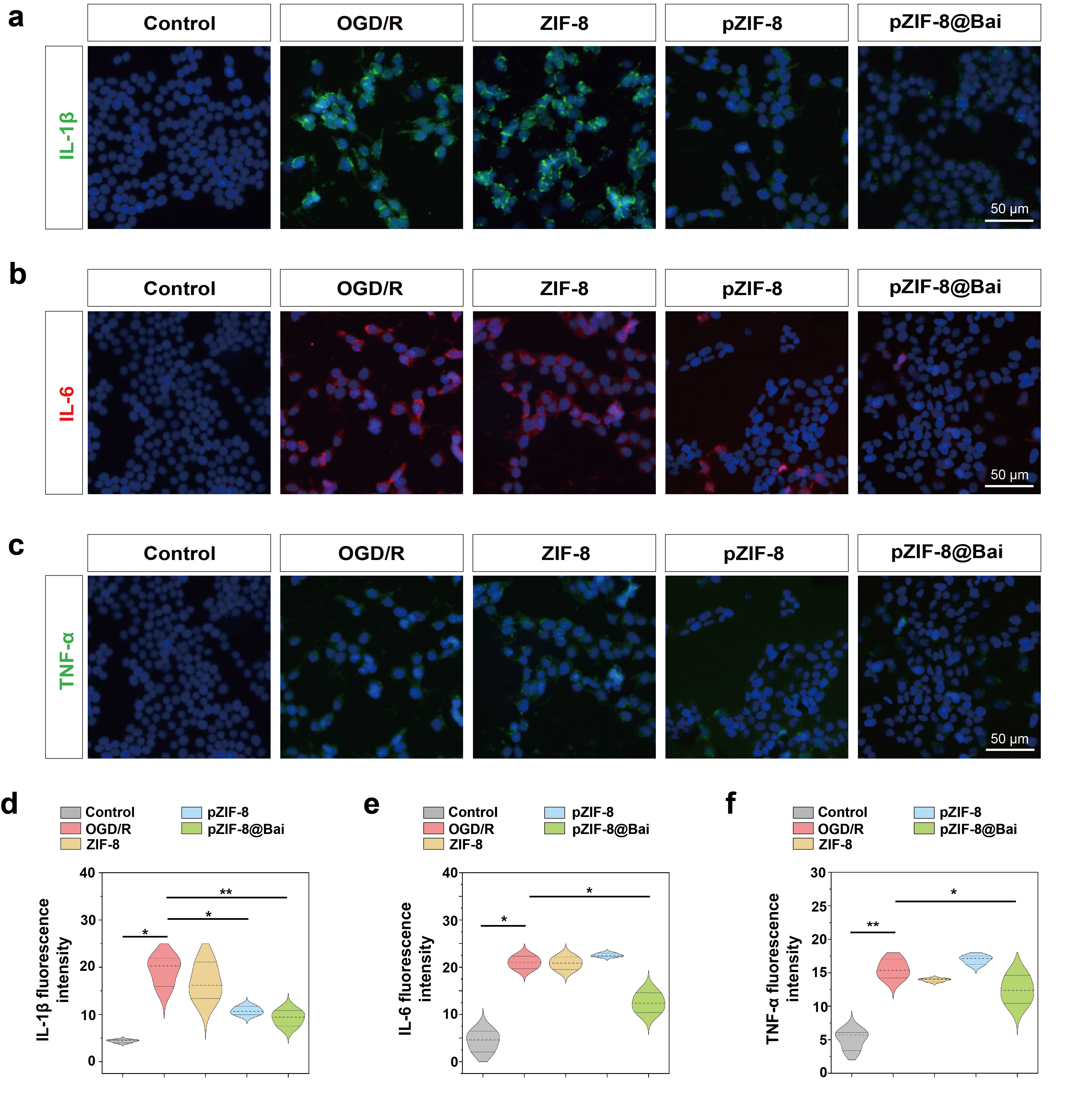


**Supplementary Figure 13.** **Representative fluorescence microscopy images of R28 cells** were stained with IL-1β a), IL-6 b), and TNF-α c) **after co-incubation with ZIF-8, pZIF-8, and pZIF-8@Bai for 24 h (25 μg/mL). Scale bar = 100 μm.** Scale bar = 50 µm. Quantification of fluorescence intensity of IL-1β d), IL-6 e), and TNF-α f) by Image J software**. Data are presented as the mean ± SD (n = 3 per group).** p values were determined by a two-tailed t-test; ns, not significant (p > 0.05); * p < 0.05, ** p < 0.01, and *** p < 0.001.

**Supplementary Figure 14. a)** Schematic of the safety evaluation in C57BL/6 mice administered with PBS or **pZIF-8@Bai** via intravitreal injection. **H&E staining b) and retinal thickness quantification c) after intravitreal injection of PBS or pZIF-8@Bai. (n = 5 mice per group).** Scale bar = 50 µm**.** d) Examination of retinal vasculature by isolectin B4 staining of whole-mount retinas. **after intravitreal injection of PBS or pZIF-8@Bai. (n = 5 mice per group).** Scale bar = 100 µm. e) **Retinal function was assessed by ERG in mice treated with PBS or pZIF-8@Bai. (n = 5 mice per group). f)** Serum levels of hepatic and renal function biomarkers in PBS- and **pZIF-8@Bai**-treated mice. **(n = 5 mice per group).** g) **Representative H&E-stained images of major organ sections obtained from mice subjected to RIR injury and various treatments for 7 days.(n = 5 mice per group).** Scale bar = 50 µm**. All results are presented as the mean ± SD.** p values (c, f) were determined by a two-tailed t-test; ns, not significant (p > 0.05); * p < 0.05, ** p < 0.01, and *** p < 0.001.**​**

**
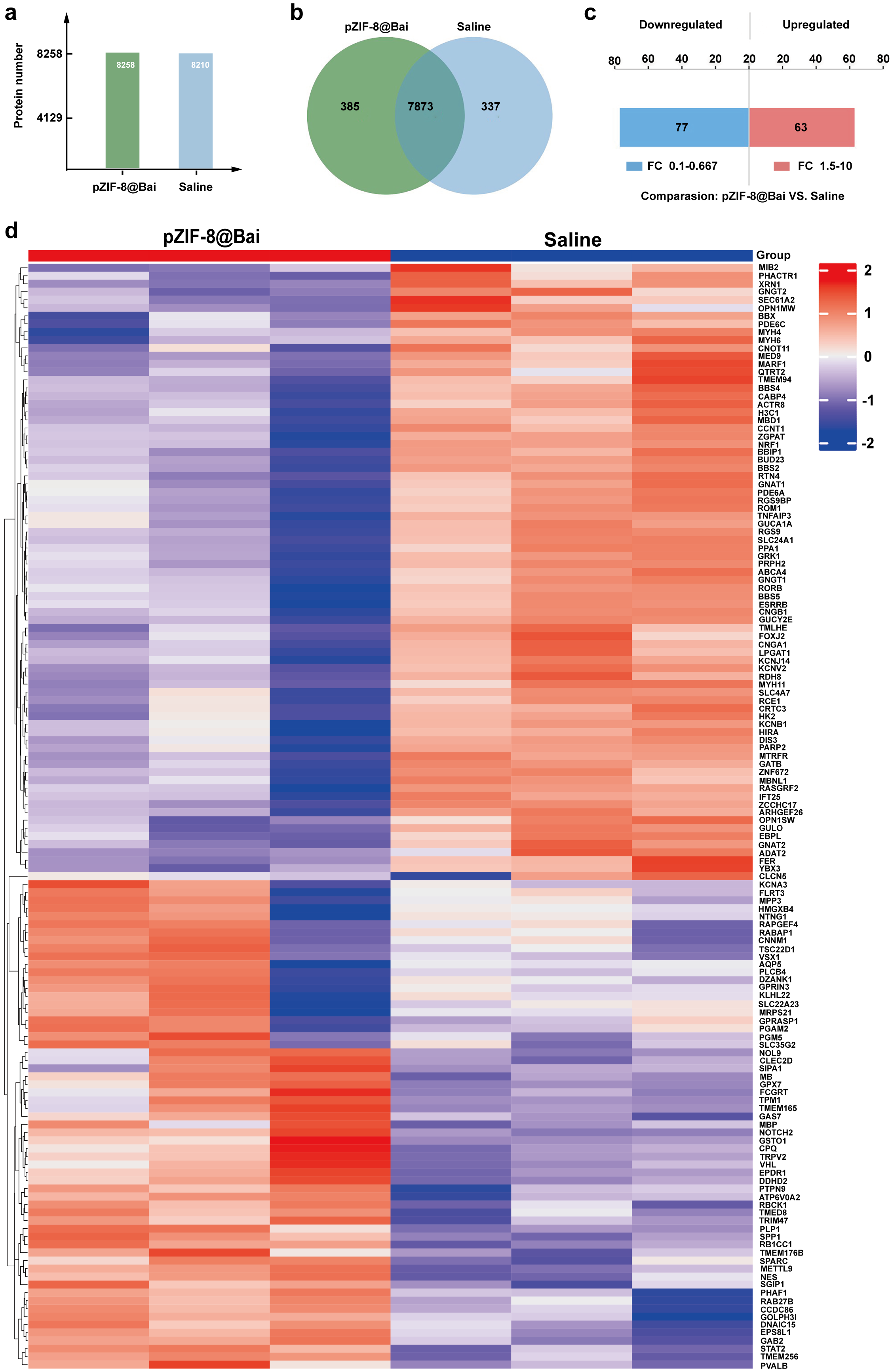
**

**Supplementary Figure 15. a) Number of protein samples identified in each group. Data are presented as the mean ± SD (n = 3 per group)**. ns, not significant (p > 0.05); * p < 0.05, ** p < 0.01, and *** p < 0.001. b) **Venn diagram of detected protein groups (saline vs.** pZIF-8@Bai**).** Numerical values represent the count of proteins in each subset. **c)** Proteomic alterations between saline and pZIF-8@Bai treatment groups (significance threshold: |FC| > 1.5, p < 0.05)**. statistically analyzed by** two-tailed t-test. d) Heat map showing the levels of shared proteins in saline, and pZIF-8@Bai groups. Red: upregulation; blue: downregulation; P value < 0.05 and fold change > 2.0.


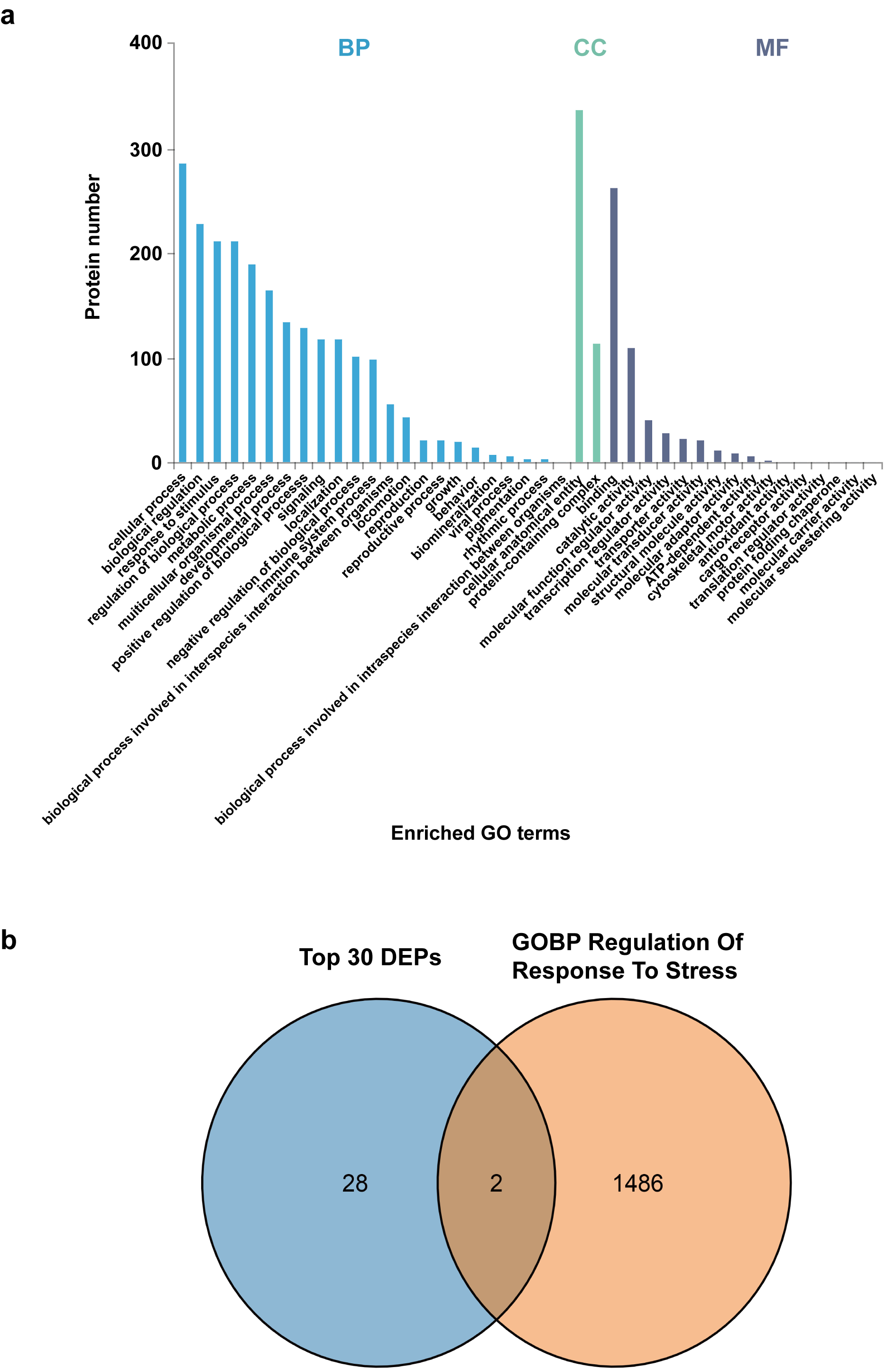


**Supplementary Figure 16. a) Histogram showing the Gene Ontology (GO) enrichment analysis results of selected differentially expressed proteins (DEPs) between the pZIF-8@Bai group and saline group. BP: biological process; CC: cellular component; MF: molecular function. (n = 3 biologically independent samples per group). Statistical significance was calculated via** a two-tailed t-test**. b)** Venn diagram of the overlap between the top 30 differentially expressed proteins and GO-defined oxidative stress pathway proteins.


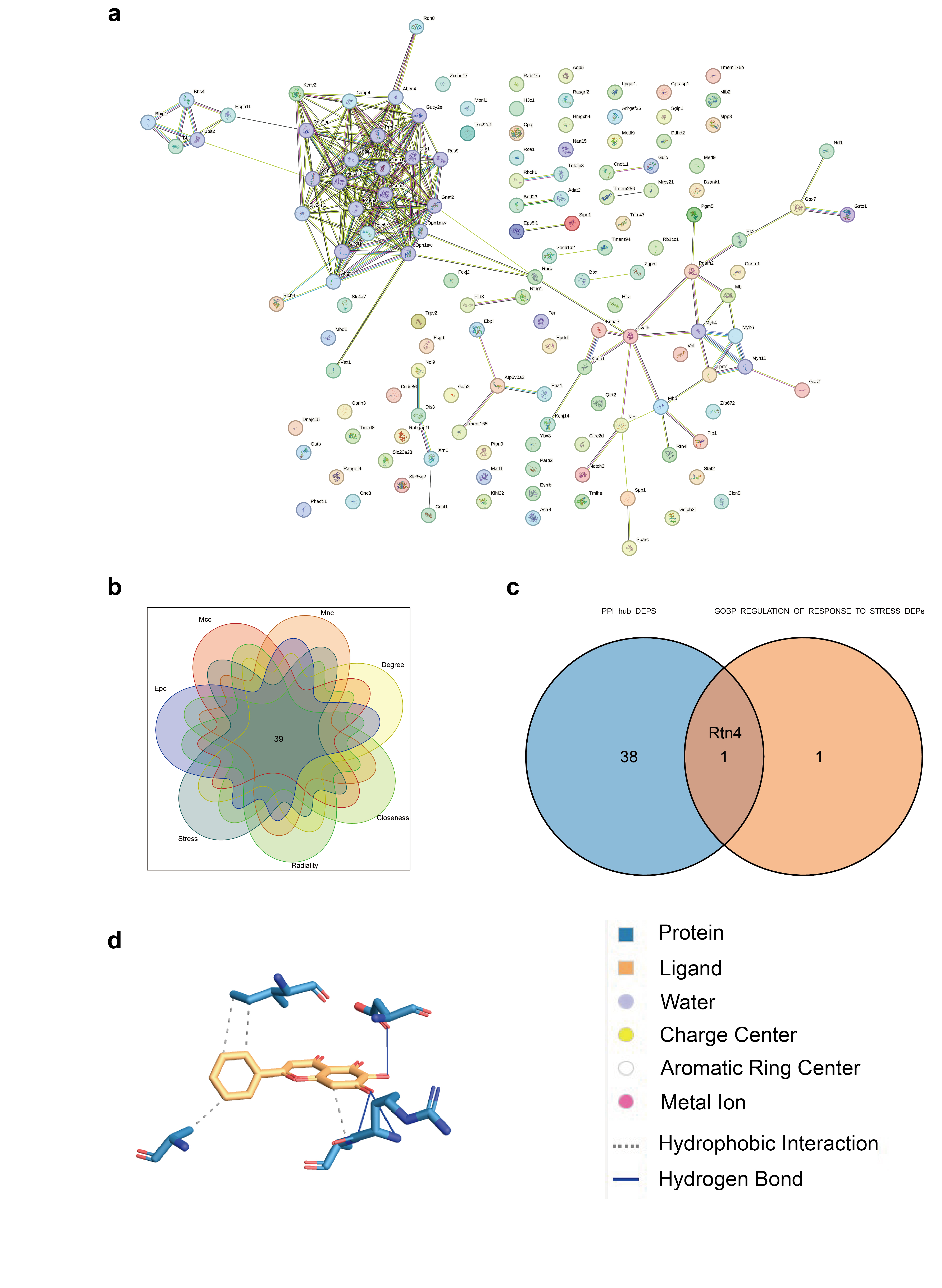


**Supplementary Figure 17 Network-based target screening and molecular docking analysis identified RTN4 as a potential key target. a) Protein-protein interaction (PPI) network of the candidate targets. Nodes represent proteins and edges represent protein-protein associations. b) Topological analysis of the PPI network using seven algorithms to identify hub targets by cytoscape. The overlapping region indicates targets with high centrality across different ranking methods. c) Venn diagram showing the intersection between hub targets obtained from the PPI network and candidate targets from the GSVA analysis. RTN4 was identified as the common target. d) Interaction model between RTN4 and the ligand. Different colors indicate protein, ligand, water, charge center, aromatic ring center, and metal ion. Gray dashed lines indicate hydrophobic interactions, and blue solid lines indicate hydrogen bonds.**


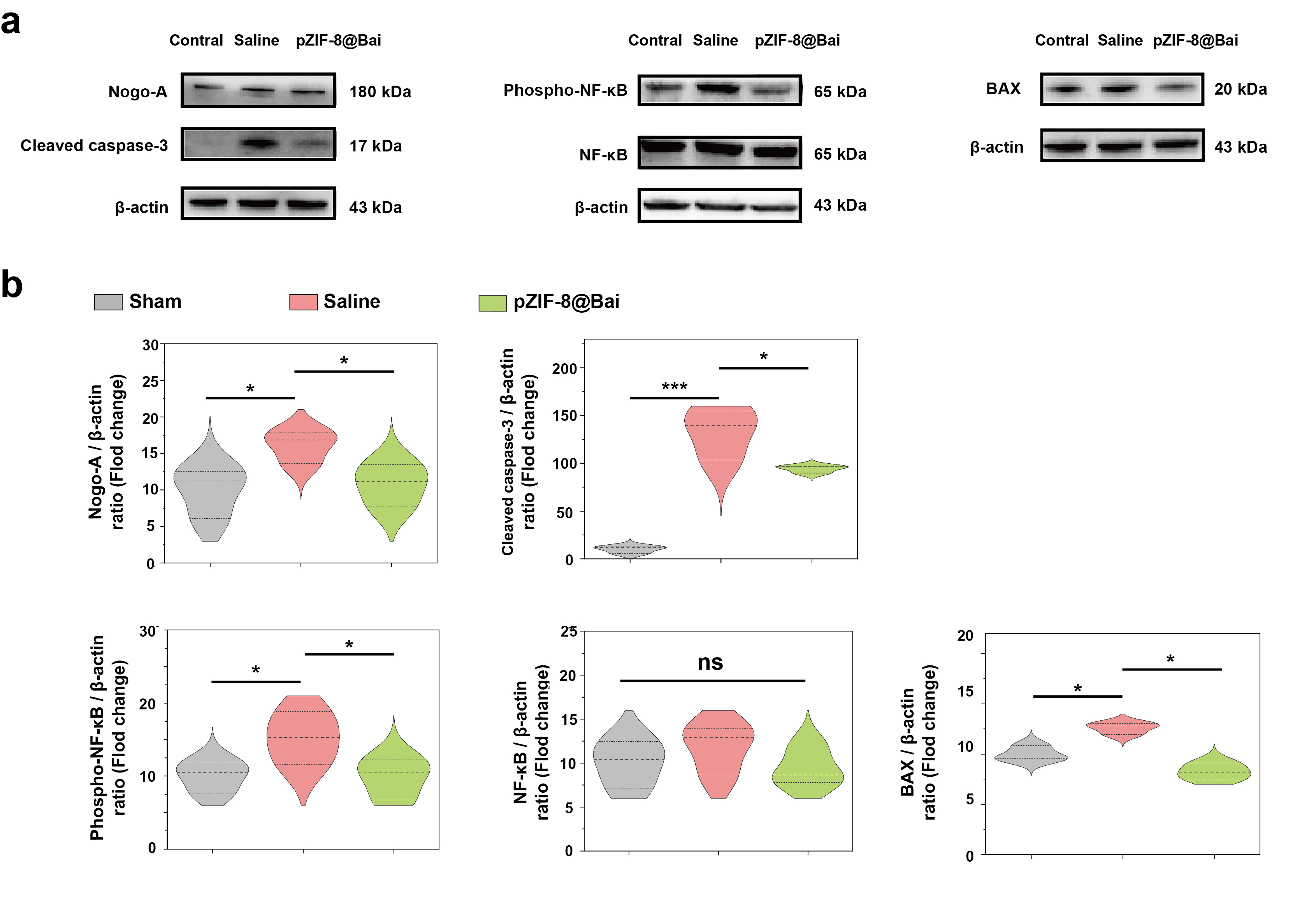


**Supplementary Figure 18.** Western blot detection of the retinal levels. Protein expression a) and quantification b) of Nogo-A, NF-κB, phospho-NF-κB, BCL-2, BAX, and cleaved caspase-3 in RIRI mice. All results are presented **as the mean ± SD (n = 3 per group).** p values (b, c, and e) were determined by one-way ANOVA employing Tukey’s post hoc test; all tests were two-sided; ns, not significant (p > 0.05); * p < 0.05, ** p < 0.01, and *** p < 0.001.

**Supplementary Table 1. Hydrophobic interactions between RTN4 and baicalein.**

| **Hydrophobic Interactions .....** | | | | | |
| --- | --- | --- | --- | --- | --- |
| **Index** | **Residue** | **AA** | **Distance** | **Ligand Atom** | **Protein Atom** |
| **4** | 28A | ALA | 3.61 | 14 | 431 |
| **3** | 21A | ALA | 3.74 | 7 | 331 |
| **2** | 10A | ILE | 3.53 | 11 | 172 |
| **1** | 10A | ILE | 3.8 | 12 | 174 |

**Supplementary Table 2.** Hydrogen Bonds **between RTN4 and baicalein.**

| **Hydrogen Bonds** | | | | | | | | | |
| --- | --- | --- | --- | --- | --- | --- | --- | --- | --- |
| Index | Residue | AA | Distance H-A | Distance D-A | Donor Angle | Protein donor? | Side  chain | Donor Atom | Acceptor Atom |
| 1 | 14A | ASP | 2.85 | 3.16 | 100.9 | √ | √ | 234［O3］ | 20［O3］ |
| 2 | 14A | ASP | 2.49 | 3.16 | 125.99 | × | √ | 20［O3］ | 234［O3］ |
| 3 | 20A | ARG | 3.68 | 4 | 101.18 | √ | × | 306［Nam］ | 19［O3］ |
| 4 | 21A | ALA | 2.55 | 3 | 107.62 | √ | × | 327［Nam］ | 19［O3］ |
